# Comparison of tanezumab and non-steroidal anti-inflammatory drugs in efficacy and safety for chronic low back pain: a systematic review and meta-analysis of randomized controlled trials

**DOI:** 10.3389/fneur.2025.1623280

**Published:** 2025-09-01

**Authors:** Dongying Yao, Shu Li, Long Pang, Pengcheng Li

**Affiliations:** ^1^Sports Medicine Center, West China Hospital, Sichuan University, Chengdu, China; ^2^Department of Orthopedics and Orthopedic Research Institute, West China Hospital, Sichuan University, Chengdu, China; ^3^West China School of Nursing, Sichuan University, Chengdu, China

**Keywords:** tanezumab, nerve growth factor (NGF) inhibitor, non-steroidal anti-inflammatory drugs (NSAIDs), chronic low back pain (CLBP), systematic review, meta-analysis

## Abstract

**Background:**

Chronic low back pain (CLBP) poses a significant global health burden often managed with non-steroidal anti-inflammatory drugs (NSAIDs). Tanezumab, a nerve growth factor (NGF) inhibitor, presents a potential alternative, but its comparative efficacy and safety relative to NSAIDs remain uncertain. This study aimed to compare tanezumab and NSAIDs for CLBP.

**Methods:**

This is a systematic review with meta-analysis, following the Preferred Reporting Items for Systematic Review and Meta-analyses (PRISMA) guidelines and Cochrane Handbook. PubMed, Excerpta Medica Database (EMBASE), the Cochrane Library, and Web of Science were searched for literature published before March 10, 2025. Outcome measures included low back pain intensity (LBPI), Roland-Morris Disability Questionnaire (RMDQ) scores, adverse events (AEs), and response rates. Manager V.5.3.3 was used for statistical assessments.

**Results:**

Three randomized controlled trials (RCTs) were included, comprising 835 participants. Tanezumab at 10 mg dose demonstrated significantly greater reduction in LBPI scores at 1-week, 4-week, 8-week, and 12-week follow-ups compared to NSAIDs. Improvement in RMDQ scores was also superior with 10 mg tanezumab at 2-week and 16-week follow-ups. The 5 mg dose, however, did not exhibit a significant difference in functional improvement compared to NSAIDs. Both the 5 mg and 10 mg tanezumab doses showed similar rates of AEs compared to NSAIDs, except for a higher incidence of abnormal peripheral sensation with 10 mg tanezumab. Response rates ≥ 50% were significantly higher with 10 mg tanezumab compared to NSAIDs.

**Conclusion:**

Tanezumab at 10 mg demonstrates better pain relief and functional improvement for CLBP compared to NSAIDs, though it increases the risk of mild peripheral sensation abnormalities. The 5 mg dose, shows a comparable safety profile but no significant therapeutic advantages. While joint safety events significantly impacted development of tanezumab for OA, their rare occurrence in peripheral joints with pre-existing OA and absence in the lumbar spine within CLBP trials suggests its risk–benefit profile appears more acceptable in CLBP than in OA.

## 1 Introduction

Chronic low back pain (CLBP), characterized by persistent pain in the lower back region lasting more than 12 weeks, is a prevalent and debilitating condition that significantly impacts the quality of life for millions worldwide (1, 2). The economic and social burden of CLBP is substantial, and disability caused by LBP has increased by 54% globally in the past two decades (3–5). The complexity of CLBP lies in its multifactorial etiology, which includes mechanical, inflammatory, neuropathic, and psychosocial components, making the identification of a single cause and subsequent targeted treatment challenging (6).

Non-steroidal anti-inflammatory drugs (NSAIDs) have long been a cornerstone of pharmacological management for CLBP, offering relief through their analgesic and anti-inflammatory properties (7–9). These drugs are widely prescribed due to their accessibility and relatively rapid onset of pain relief (10). However, the use of NSAIDs is not without risk, as they are associated with a range of adverse effects, including gastrointestinal complications, cardiovascular events, and renal impairment, especially for older people (11, 12). There is a need for safer and more effective therapeutic alternatives for the management of CLBP.

The 1986 Nobel Prize in Physiology and Medicine was awarded to Rita Levi-Montalcini and Stanley Cohen for their 1950s research on nerve growth factor (NGF) and its role in peripheral nervous system development. NGF is part of a family of proteins, including brain-derived neurotrophic factor (BDNF) and neurotrophic factor 3 (NT-3), promoting the survival and growth of peripheral nerves (13). It modulates nociceptive processing through various mechanisms such as sensitization, altered transcriptional regulation, and increased innervation in diseased connective tissue. NGF binds to tropomyosin-related kinase-A (Trk-A), triggering downstream signaling pathways that influence pain development and maintenance (13–15). NGF’s involvement in CLBP is complex, potentially contributing to both nociceptive and neuropathic components (16, 17). Preclinical studies suggest NGF’s role in lumbar degenerative disk disease (DDD) and facet joint pain (18, 19). However, its precise role in other lumbar and cervical spine conditions remains unclear due to limited research.

Recently, anti-NGF monoclonal antibodies (mAbs) have emerged as a novel class of medications being developed to treat various chronic painful conditions (20). While extensively studied in clinical trials for osteoarthritis (OA) affecting the hips and knees, the development of tanezumab was ultimately suspended primarily due to significant safety concerns, most notably rapidly progressive osteoarthritis (RPOA) and osteonecrosis (21–26). Indeed, multiple Phase 3 OA trials (27–30) and pooled analyses (31–33) have consistently revealed that adjudicated composite joint safety events (CJSEs), predominantly RPOA, occurred more frequently in tanezumab-treated groups compared to placebo or NSAIDs, exhibiting a dose-dependent risk.

Despite safety concerns, tanezumab, an NGF-inhibiting mAb, demonstrates superior efficacy over placebo patients with CLBP (21, 34–37). Notably, in CLBP patients, its safety profile primarily highlights mild-to-moderate abnormal peripheral sensation rather than severe joint issues (21, 26). However, a direct comparative evaluation of tanezumab against NSAIDs concerning both efficacy and safety for CLBP remains largely unaddressed. This study aims to bridge this critical gap by comprehensively comparing tanezumab and NSAIDs for CLBP management, with a particular focus on their respective safety profiles.

This is the first systematic review and meta-analysis aimed to comprehensively assess tanezumab’s efficacy and safety relative to NSAIDs in the treatment of CLBP. By synthesizing existing evidence, we aim to inform clinical decision-making, refine treatment guidelines, and enhance patient care. We hypothesize that while both tanezumab and NSAIDs offer benefits for CLBP, tanezumab’s efficacy may be dose-dependent, potentially outperforming NSAIDs at higher doses.

## 2 Methods

### 2.1 Study design and ethical framework

This is a systematic review with meta-analysis, evaluating the efficacy and safety of tanezumab versus NSAIDs for CLBP, following the Preferred Reporting Items for Systematic Review and Meta-analyses (PRISMA) guidelines and Cochrane Handbook for Systematic Reviews of Interventions standards (50). As part of a broader chronic pain research project at West China Hospital, Sichuan University, the predefined methodological framework and protocol was submitted to the hospital’s Medical Ethics Committee for ethical approval.

### 2.2 Focused question

This systematic review with meta-analysis was guided by a predefined PICO (Population, Intervention, Comparison, Outcome) framework. Our framework defined the Population as adult patients with CLBP of at least duration of 3 months; the Intervention as tanezumab; the Comparison as oral NSAIDs; and the Outcomes as pain intensity, functional improvement, adverse events (AEs), and response rates.

### 2.3 Search strategy

The literature search was conducted ethically, ensuring transparency, reproducibility, and impartiality based on the PICO framework. Two independent researchers (D. Y. and S. L.) individually performed searches on PubMed, Excerpta Medica Database (EMBASE), the Cochrane Library, and Web of Science. The searches were done using the specified search terms: (Tanezumab OR nerve growth factor OR NGF OR anti-NGF OR NGF antibody) AND (non-steroidal anti-inflammatory drugs OR NSAIDs) AND (low back pain OR chronic low back pain OR low back pain, chronic OR LBP OR CLBP). Only studies published on or before March 10, 2025 were considered for inclusion in this systematic review. All potentially qualifying studies comparing tanezumab and NSAIDs for patients with CLBP were obtained manually. Any contentious disagreement was addressed with the intervention of a third researcher (P. L.).

### 2.4 Selection criteria

The inclusion criteria are as follows: (1) patients were adults who had a confirmed diagnosis of CLBP at least 3 months; (2) patients in one group received tanezumab whereas patients in another group received NSAIDs; (3) the study design must be a randomized controlled trial (RCT); (4) at least one of the following outcomes was reported: low back pain intensity (LBPI) (38), Roland-Morris Disability Questionnaire (RMDQ) (39); and (5) the incidence of AEs or safety data were reported as mandatory outcomes.

The exclusion criteria are as follows: (1) non-randomized or observational studies; (2) studies that enrolled participants with other types of chronic pain; (3) studies not written in English.

### 2.5 Data extraction

Data from the studies included in the analysis was collected independently by two researchers (D. Y. and S. L.). All relevant information from the selected studies, including the first author, year, national clinical trial number (NCT), trial phase, region, follow-up, and study arms, as well as baseline information on the patients such as age, gender, race, body mass index (BMI), duration of CLBP, primary etiology, baseline LBPI and RMDQ scores, were thoroughly recorded. The study collected and combined the following clinical outcomes: (1) change from baseline (*Δ*) in LBPI score; (2) Δ RMDQ score; (3) AEs, including any AE, serious AE, treatment discontinuation due to AE, abnormal peripheral sensation and joint safety events; and (4) the proportions of response rate ≥ 30% and response rate ≥ 50% among different groups. Response rate was defined as the proportion of patients achieving a predefined percentage reduction in improvement in LBPI from baseline.

### 2.6 Quality assessment

**Figure 1:**
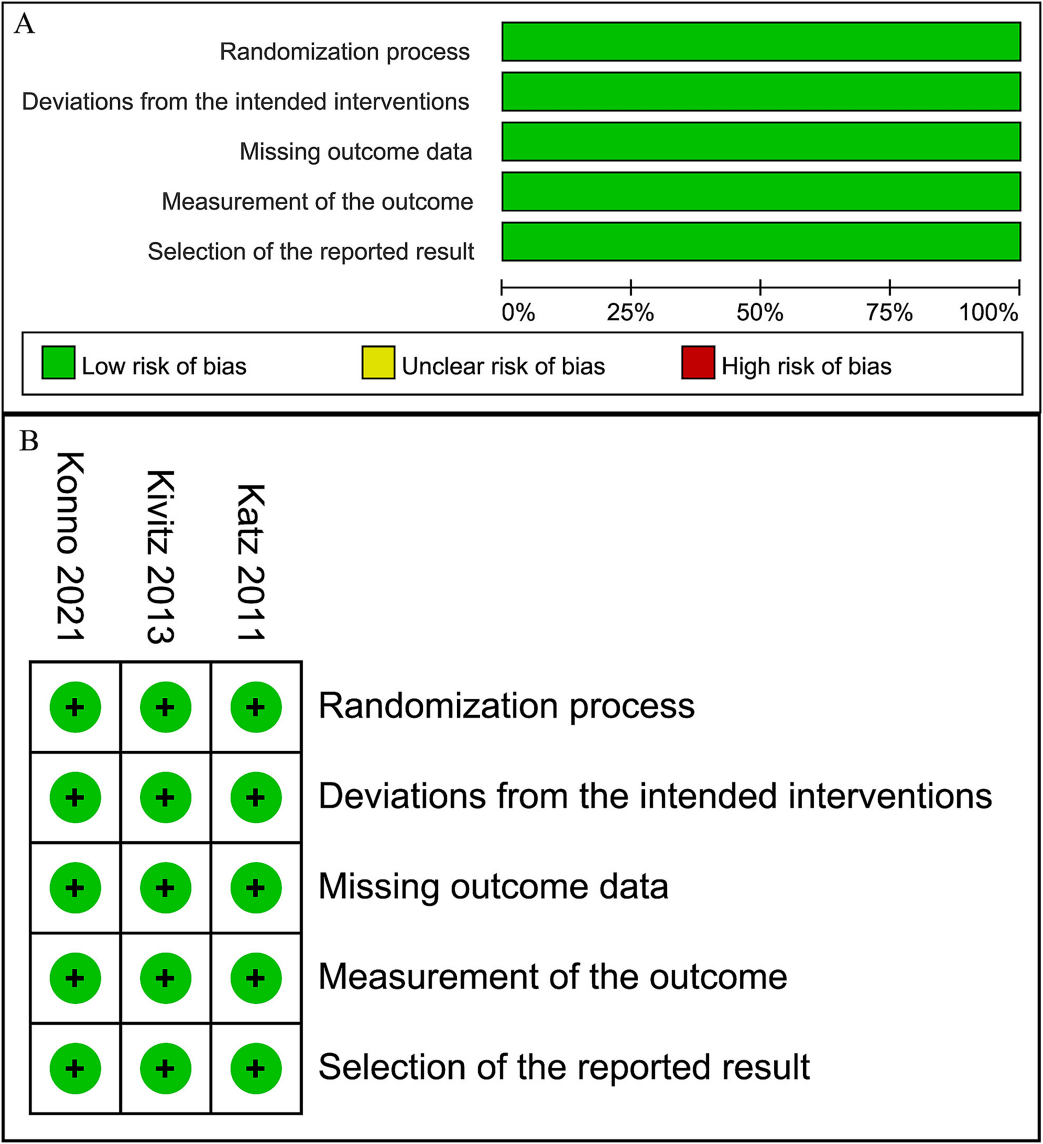
Risk of bias graph. **(A)** Graph of the risk of bias summary for the included studies, **(B)** graph of the risk of bias for each included study.

Two researchers (D. Y. and S. L.) assessed the methodological quality of included studies independently, using the revised Cochrane Risk of Bias 2 (RoB 2) instrument for RCTs (40). Discussions were carried out with a third author (P. L.) to solve any disagreements. The resulting risk of bias graphs (Figure 1) were then generated using Review Manager (RevMan) V.5.3.3 (The Cochrane Collaboration, Software Update, Oxford, United Kingdom). Publication bias was not studied as the number of publications in each study field was fewer than 10.

### 2.7 Data synthesis and analysis

RevMan V.5.3.3was used for statistical assessments. To assess the outcomes, we calculated the weighted mean difference (WMD) and pooled odds ratio (OR) with corresponding 95% confidence intervals (CIs). A *p*-value greater than 0.05 was considered statistically significant. We analyzed the heterogeneity of each qualifying trial using Cochrane’s *Q* and *I^2^* statistics. When the heterogeneity is considerable (*I^2^* is higher than 50%), the data will be synthesized using a random-effect model, otherwise a fixed-effect model will be employed. Due to the limited number of included studies, subgroup analyses were not performed.

## 3 Results

### 3.1 Search results and study characteristics

**Figure 2:**
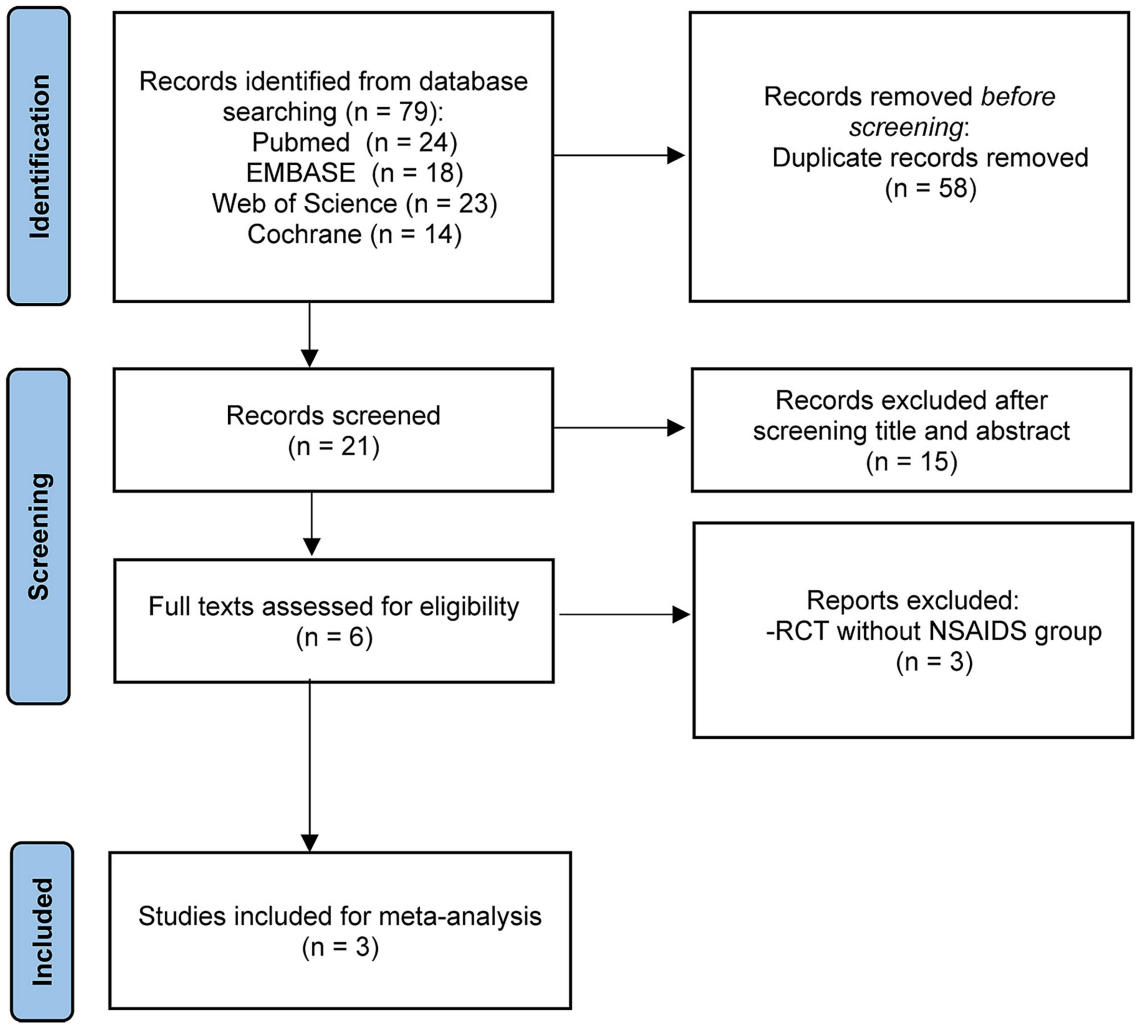
PRISMA flow chart.

Following searches on PubMed, EMBASE, the Cochrane Library, and Web of Science by two independent researchers using predefined search terms, a total of 79 articles available until March 10, 2025 were retrieved. After removing 58 duplicates, the remaining 21 articles underwent screening of titles and abstracts, leading to the exclusion of 15 articles. Subsequently, the full texts and references of the remaining 6 articles were screened, resulting in the exclusion of 3 RCTs (41–43) based on the selection criteria. Ultimately, 3 studies (44–46) were included in this meta-analysis (Figure 2). The detailed characteristics of the enrolled studies and patients are presented in Tables 1, 2.

**Table 1:** Characteristics of the included studies.

Study	NCT	Trial phase	Region	Follow-up	Study arms
Katz et al. (46)	NCT00924664	II	Multicenter in USA	16 weeks	Single IV infusion tanezumab 200 μg/kg (*n* = 88); Oral naproxen 500 mg b.i.d. daily (*n* = 88); Placebo (*n* = 41)
Inclusion criteria	(1) Age >18 years and BMI ≤ 39 kg/m^2^; (2) CLBP ≥3 months requiring regular use of analgesic medication (>4 days/week for the past month); (3) Primary location of low back pain is between the 12th thoracic vertebra and the lower gluteal folds, with or without radiation into the posterior thigh, classified as category 1 or 2 according to the classification of the Quebec Task Force in Spinal Disorders; (4) Must have a score of ≥4 for LBPI while on current treatment at screening and completes at least 4 daily pain diaries during the 5 d before randomization, with an average LBPI score of ≥4.				
Exclusion criteria	(1) Lumbosacral radiculopathy within the past 2 years; (2) Spinal stenosis associated with neurological impairment or neurogenic claudication; (3) Back pain due to visceral disorder; (4) Patients with history, diagnosis, or signs and symptoms of clinically significant neurological disease, recent major trauma, osteoporotic compression fracture, or surgical intervention for the treatment of LBP; (5) Pregnancy or lactation; (6) Rheumatoid arthritis; (7) Seronegative spondyloarthropathy; (8) Paget disease of the spine, pelvis, or femur; fibromyalgia; (9) Tumors or infections of the spinal cord; (10) Cancer within the last 2 years, other than cutaneous basal cell or squamous cell carcinoma resolved by excision; (11) Allergic or anaphylactic reaction to a therapeutic or diagnostic monoclonal antibody or IgG fusion protein; (12) Intolerance to acetaminophen or any of its excipients; (13) Hypersensitivity to NSAIDs or any condition that might have precluded the use of an NSAID; (14) Clinically significant cardiovascular, chronic viral, or neurological disease; (15) Psychiatric disorders.				
Kivitz et al. (45)	NCT00876187	II	Multicenter in USA	16 weeks	IV Tanezumab 5 mg at baseline and week 8 (*n* = 232); IV Tanezumab 10 mg at baseline and week 8 (*n* = 295); IV Tanezumab 20 mg at baseline and week 8 (*n* = 295); Naproxen 500 mg b.i.d. daily (*n* = 295); Placebo (*n* = 230)
Inclusion criteria	(1) CLBP ≥3 months requiring regular use of analgesic medication (>4 days/week for the past month) including immediate-release opioids (in which the average daily opioid dose [for a 7-day period] did not exceed a morphine equivalent dose of 30 mg/day) but excluding acetaminophen, gabapentin, or pregabalin as the sole analgesics used for CLBP; (2) Primary location of low back pain between the 12th thoracic vertebra and the lower gluteal folds, with or without radiation into the posterior thigh (Quebec Task Force on Spinal Disorders category 1 or 2); (3) Average LBPI score of ≥4 while receiving current treatment; (4) Patient’s Global Assessment of Low Back Pain of fair, poor, or very poor.				
Exclusion criteria	(1) Lumbosacral radiculopathy within the past 2 years, vertebral fracture, major trauma, or back surgery in the past 6 months; (2) Significant cardiac, neurological, or other pain, or psychological conditions; (3) Known history of rheumatoid arthritis, seronegative spondyloarthropathy; (4) Paget disease of the spine, pelvis, or femur; (5) Fibromyalgia; tumors or infections of the spinal cord; (6) Any condition that might preclude NSAID use; (7) Patients also were excluded if extended-release opioids or long-acting opioids such as oxycodone controlled-release, oxymorphone extended-release, hydromorphone, transdermal fentanyl, or methadone had been used within 3 months of screening.				
Konno et al. (44)	NCT02725411	III	Multicenter in Japan	80 weeks	SC Tanezumab 5 mg every 8 weeks (*n* = 92); SC Tanezumab 10 mg every 8 weeks (*n* = 93); Celecoxib 100 mg b.i.d. daily (*n* = 92)
Inclusion criteria	(1) Patients aged ≥18 years with CLBP (primary location between the 12th thoracic vertebra and lower gluteal folds, with or without radiation into the posterior thigh [category 1 or 2 per Quebec Task Force in Spinal Disorders]) of ≥3 months’ duration; (2) LBPI score ≥5 at screening and baseline; (3) Patient’s Global Assessment of Low Back Pain score of fair, poor, or very poor at baseline; (4) Patients were required to be experiencing some benefit from, and ability to tolerate, a stable (≥5 days/week in the 30 days prior to baseline) regimen of oral NSAID therapy (celecoxib 100 mg twice daily, loxoprofen 120–180 mg/day, or meloxicam 5–15 mg/day) but still require additional pain relief at screening.				
Exclusion criteria	(1) A history of lumbosacral radiculopathy; (2) Diagnosis of osteoarthritis of the knee or hip based on American College of Rheumatology combined clinical and radiographic criteria, KL-based radiographic evidence of hip (grade ≥2) or knee (grade ≥3) osteoarthritis; (3) Radiographic evidence and symptoms of osteoarthritis of the shoulders.				

NCT, national clinical trial number; IV, intravenous; SC, subcutaneous; b.i.d., twice daily; BMI, body mass index; CLBP, chronic low back pain; LBPI, low back pain intensity; NSAIDs, non-steroidal anti-inflammatory drugs; KL, Kellgren–Lawrence.

**Table 2:** Baseline characteristics of included patients*.

Included studies and arms								
Characteristic	Katz et al. (46)		Kivitz et al. (45)			Konno et al. (44)		
	Tanezumab 200 μg/kg (*n* = 88)	Naproxen 500 mg b.i.d. (*n* = 88)	Tanezumab 5 mg (*n* = 232)	Tanezumab 10 mg (*n* = 295)	Naproxen 500 mg b.i.d. (*n* = 295)	Tanezumab 5 mg (*n* = 92)	Tanezumab 10 mg (*n* = 93)	Celecoxib 100 mg b.i.d. (*n* = 92)
Age, mean ± SD, y	49.5 ± 14.7	52.1 ± 14.8	51.5 ± 11.7	52 ± 11.0	52.6 ± 11.5	53.3 ± 14.25	52.3 ± 14.25	54.3 ± 15.5
Gender, *n* (%)								
Male	35 (39.8)	46 (52.3)	117 (50.4)	138 (46.8)	143 (48.5)	55 (59.8)	49 (52.7)	54 (58.7)
Female	53 (60.2)	42 (47.7)	115 (49.6)	157 (53.2)	152 (51.5)	37 (40.2)	44 (47.3)	38 (41.3)
Race, *n* (%)								
White	81 (92.0)	82 (93.2)	187 (80.6)	238 (80.7)	224 (75.9)	0 (0)	0 (0)	0 (0)
Black	1 (1.1)	5 (5.7)	32 (13.8)	43 (14.6)	57 (19.3)	0 (0)	0 (0)	0 (0)
Asian	2 (2.3)	0	4 (1.7)	5 (1.7)	3 (1.0)	92 (100)	93 (100)	92 (100)
Other	4 (4.5)	1 (1.1)	9 (3.9)	9 (3.1)	11 (3.7)	0 (0)	0 (0)	0 (0)
BMI, mean ± SD, kg/m^2^	28.8 ± 4.8	28.6 ± 4.8	29.2 ± 4.9	29.3 ± 4.9	30.3 ± 5.0	24.1 ± 3.9	23.9 ± 4.2	23.9 ± 3.6
Duration of CLBP, mean (range), y	10.0 (0.3–48.3)	13.0 (0.4–52.9)	10.9 (0.3–67.7)	11.2 (0.4–55.9)	11.2 (0.3–53.9)	8.9 (8.8)	7.7 (9.1)	9.1 (10.5)
Primary etiology, *n* (%)								
Disk disease	30 (34.1)	21 (23.9)	64 (27.6)	82 (27.8)	69 (23.4)	32 (34.8)	40 (43.0)	38 (41.3)
Degenerative joint disease/OA	33 (37.5)	37 (42.0)	90 (38.8)	98 (33.2)	125 (42.4)	13 (14.1)	11 (11.8)	17 (18.5)
Injury/muscular strain	21 (23.9)	20 (22.7)	73 (31.5)	109 (36.9)	96 (32.5)	6 (6.5)	0	1 (1.1)
Other	4 (4.5)	10 (11.4)	5 (2.2)	6 (2.0)	5 (1.7)	41 (44.6)	42 (45.2)	36 (39.1)
LBPI, mean ± SD	6.5 ± 1.4	6.7 ± 1.4	6.62 ± 1.4	6.57 ± 1.4	6.77 ± 1.4	6.74 ± 0.97	6.82 ± 1.09	6.72 ± 1.00
RMDQ, mean ± SD	12.3 ± 4.6	12.4 ± 4.8	12.24 ± 4.9	12.98 ± 5.1	12.86 ± 4.9	8.27 ± 5.02	8.12 ± 4.86	7.75 ± 4.95

y, years; SD, standard deviation; b.i.d., twice daily; BMI, body mass index; CLBP, chronic low back pain; OA, osteoarthritis; LBPI, low back pain intensity; RMDQ, Roland–Morris Disability Questionnaire score.

*Only the study arms that were included in meta-analyses are represented in this table.

### 3.2 Risk of bias in included studies

The risk of bias in the included RCTs is depicted in Figure 1. All the included RCTs are of high quality, with a low risk in each domain of RoB2 assessment.

### 3.3 Low back pain intensity (LBPI)

**Figure 3:**
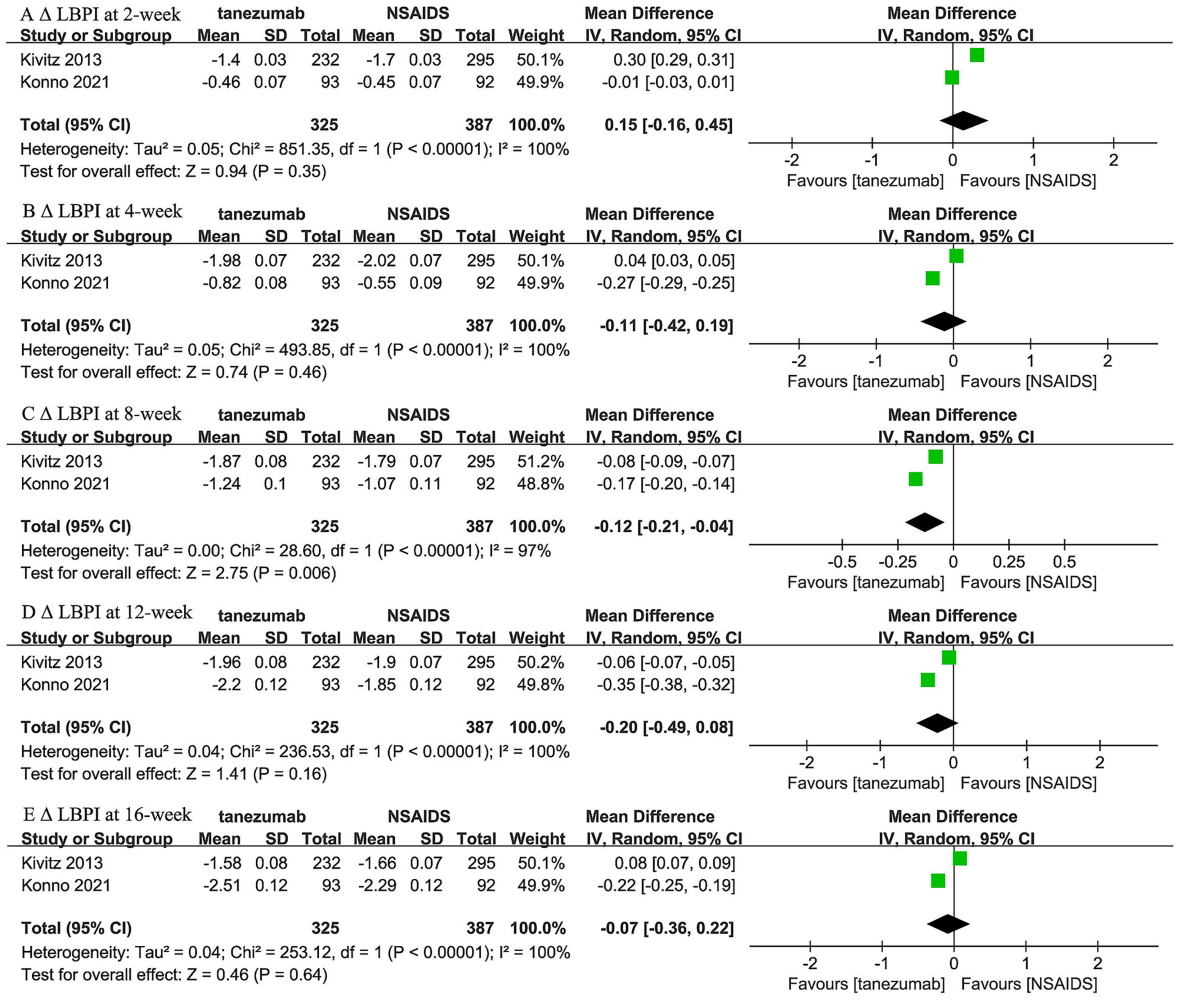
Meta-analysis of 5 mg tanezumab versus NSAIDs in *Δ* LBPI scores at **(A)** 2-week, **(B)** 4-week, **(C)** 8-week, **(D)** 12-week, and **(E)** 16-week follow-ups.

Two trials (44, 45) reported the comparison between 5 mg tanezumab and NSAIDs in *Δ* LBPI scores at 2-week, 4-week, 8-week, 12-week, and 16-week follow-ups (Figure 3). No statistical differences in Δ LBPI scores were identified between the 5 mg tanezumab and NSAIDs groups at 2-week (WMD, 0.15; 95% CI, −0.16 to 0.45; *I^2^* = 100%; *p* = 0.35), 4-week (WMD, −0.11; 95% CI, −0.42 to 0.19; *I^2^* = 100%; *p* = 0.46), 12-week (WMD, −0.20; 95% CI, −0.49 to 0.08; *I^2^* = 100%; *p* = 0.16), or 16-week (WMD, −0.07; 95% CI, −0.36 to 0.22; *I^2^* = 100%; *p* = 0.64) follow-ups. However, at the 8-week follow-up, the 5 mg tanezumab group exhibited a statistically significant reduction in *Δ* LBPI score compared to the NSAIDs group (WMD, −0.12; 95% CI, −0.21 to −0.04; *I^2^* = 97%; *p* = 0.006).

**Figure 4:**
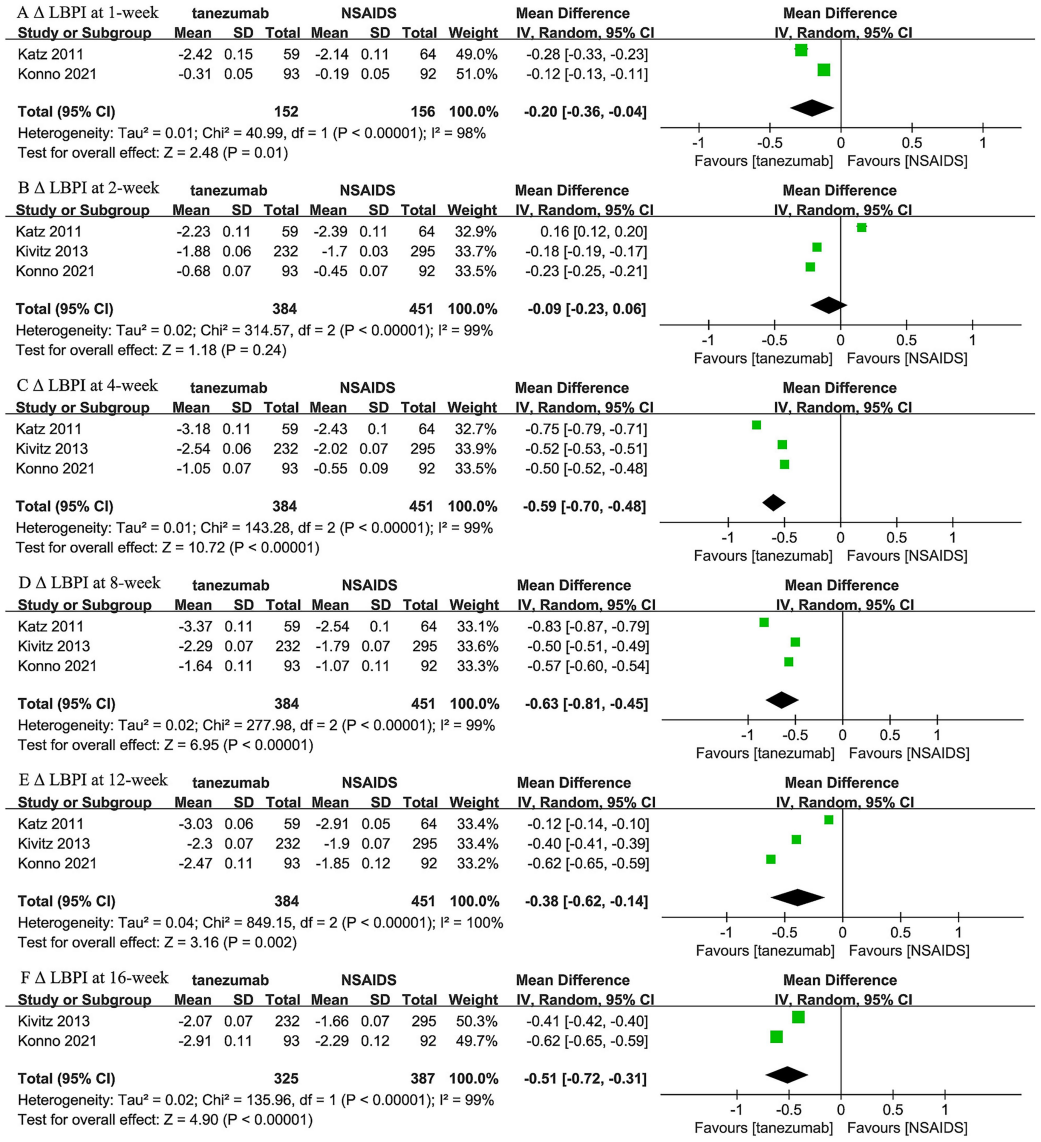
Meta-analysis of 10 mg tanezumab versus NSAIDs in Δ LBPI scores at **(A)** 1-week, **(B)** 2-week, **(C)** 4-week, **(D)** 8-week, **(E)** 12-week, and **(F)** 16-week follow-ups.

Regarding the comparison between 10 mg tanezumab and NSAIDs, two trials (44, 46) reported 1-week and 16-week follow-up, while three trials reported results for 2-week, 4-week, 8-week, and 12-week follow-ups regarding Δ LBPI scores (Figure 4). A comparable reduction in LBPI score was observed only at the 2-week follow-up (WMD, −0.09; 95% CI, −0.23 to 0.06; *I^2^* = 99%; *p* = 0.24). However, the 10 mg tanezumab group demonstrated a significantly superior reduction compared to the NSAIDs group in LBPI score at 1-week (WMD, −0.20; 95% CI, −0.36 to −0.04; *I^2^* = 98%; *p* < 0.001), 4-week (WMD, −0.59; 95% CI, −0.70 to −0.48; *I^2^* = 99%; *p* < 0.001), 8-week (WMD, −0.63; 95% CI, −0.81 to −0.45; *I^2^* = 99%; *p* < 0.001), 12-week (WMD, −0.38; 95% CI, −0.62 to −0.14; *I^2^* = 100%; *p* = 0.002), and 16-week (WMD, −0.09; 95% CI, −0.23 to 0.06; *I^2^* = 99%; *p* < 0.001) follow-ups.

### 3.4 Roland–Morris disability questionnaire (RMDQ)

**Figure 5:**
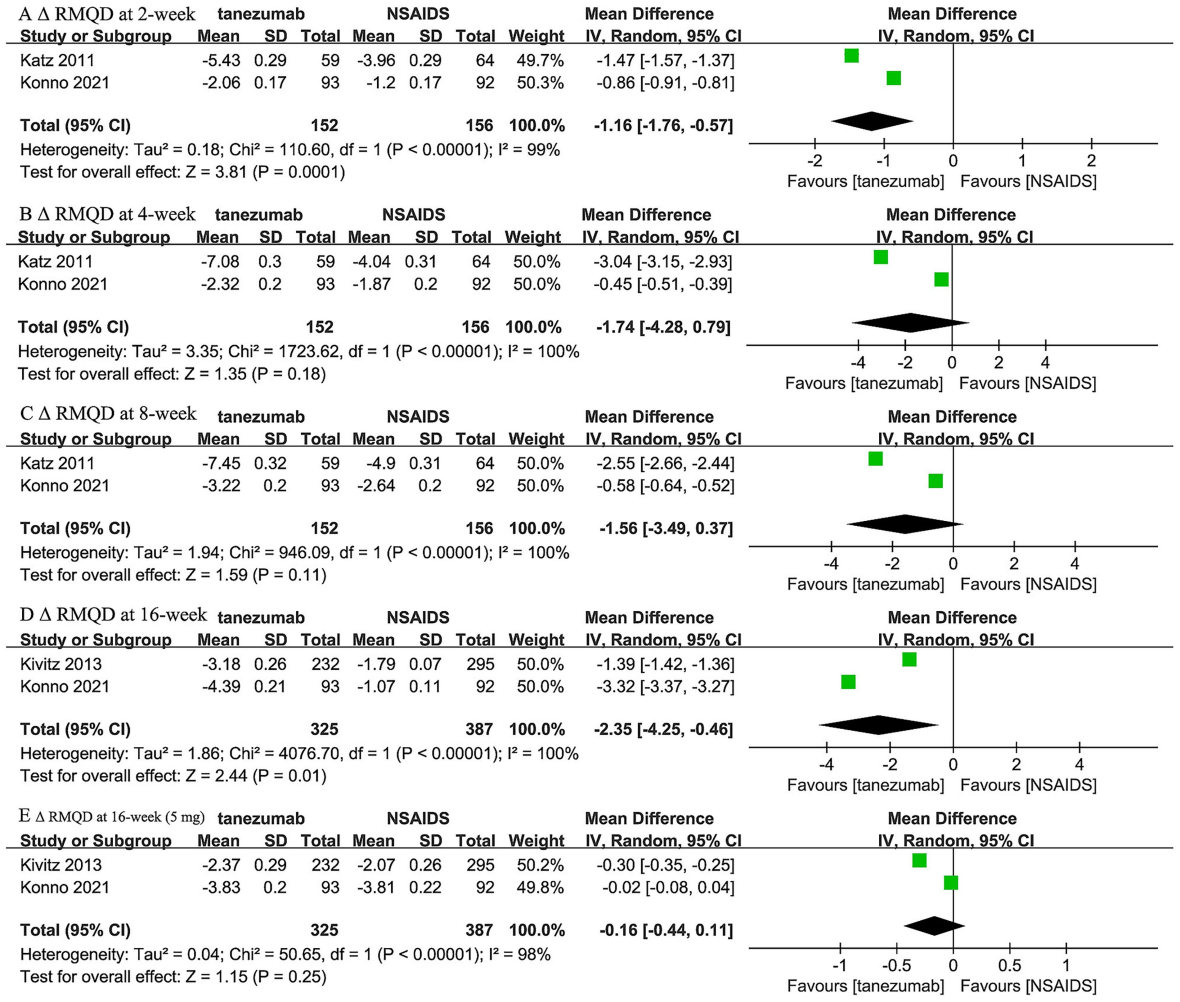
Meta-analysis of 10 mg tanezumab versus NSAIDs in Δ RMDQ scores at **(A)** 2-week, **(B)** 4-week, **(C)** 8-week, **(D)** 16-week; 5 mg tanezumab versus NSAIDs in Δ RMDQ scores at **(E)** 16-week.

Two trials (44, 46) reported the comparison between 10 mg tanezumab and NSAIDs in *Δ* RMDQ scores at 2-week, 4-week, and 8-week intervals, while two trials (44, 45) reported both 5 mg and 10 mg tanezumab versus NSAIDs at the 16-week follow-up in Δ RMDQ scores (Figure 5). Compared to the NSAIDs group, the 10 mg tanezumab group exhibited statistically superior Δ RMDQ scores at 2-week (WMD, −1.16; 95% CI, −1.76 to −0.57; *I^2^* = 99%; *p* = 0.0001) and 16-week (WMD, −2.35; 95% CI, −4.25 to −0.46; *I^2^* = 100%; *p* = 0.01) follow-ups. However, similar Δ RMDQ scores were observed at the 4-week (WMD, −1.74; 95% CI, −4.28 to 0.79; *I^2^* = 100%; *p* = 0.18) and 8-week (WMD, −1.56; 95% CI, −3.49 to 0.37; *I^2^* = 100%; *p* = 0.11) follow-ups. In contrast, the 5 mg tanezumab group failed to demonstrate a greater reduction in RMDQ score than the NSAIDs group (WMD, −0.16; 95% CI, −0.44 to 0.11; *I^2^* = 98%; *p* = 0.25) at 16-week follow-up.

### 3.5 Adverse events (AEs)

**Figure 6:**
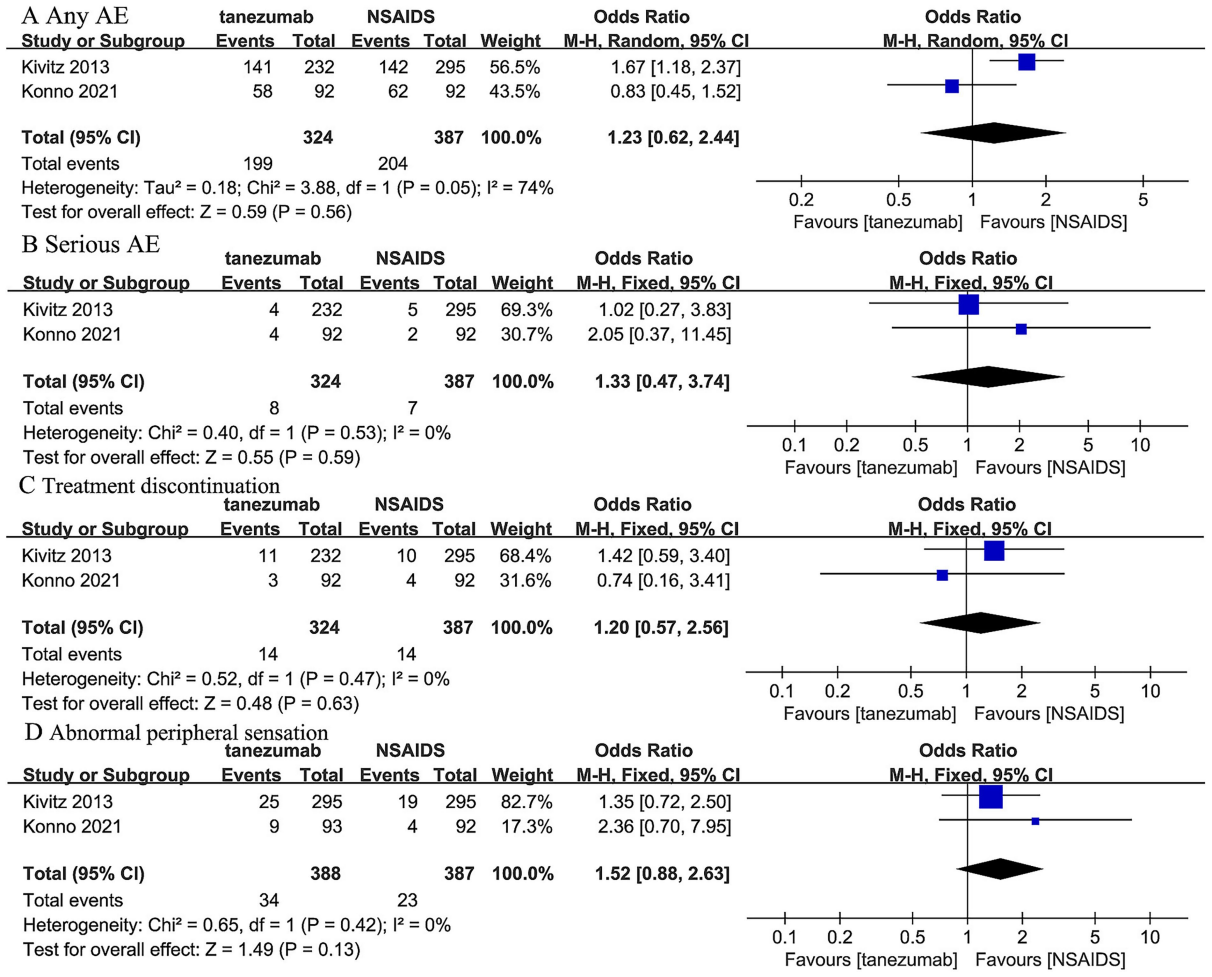
Meta-analysis of 5 mg tanezumab versus NSAIDs in **(A)** any AE, **(B)** serious AE, **(C)** treatment discontinuation due to AE, **(D)** abnormal peripheral sensation.

Two trials (44, 45) reported that 5 mg tanezumab and NSAIDs exhibited similar results in any AE (OR, 1.23; 95% CI, 0.62 to 2.44; *I^2^* = 74%; *p* = 0.56), serious AE (OR, 1.33; 95% CI, 0.47 to 3.74; *I^2^* = 0%; *p* = 0.59), treatment discontinuation due to AE (OR, 1.20; 95% CI, 0.57 to 2.56; *I^2^* = 0%; *p* = 0.63), and abnormal peripheral sensation (OR, 1.52; 95% CI, 0.88 to 2.63; *I^2^* = 0%; *p* = 0.13) (Figure 6).

**Figure 7:**
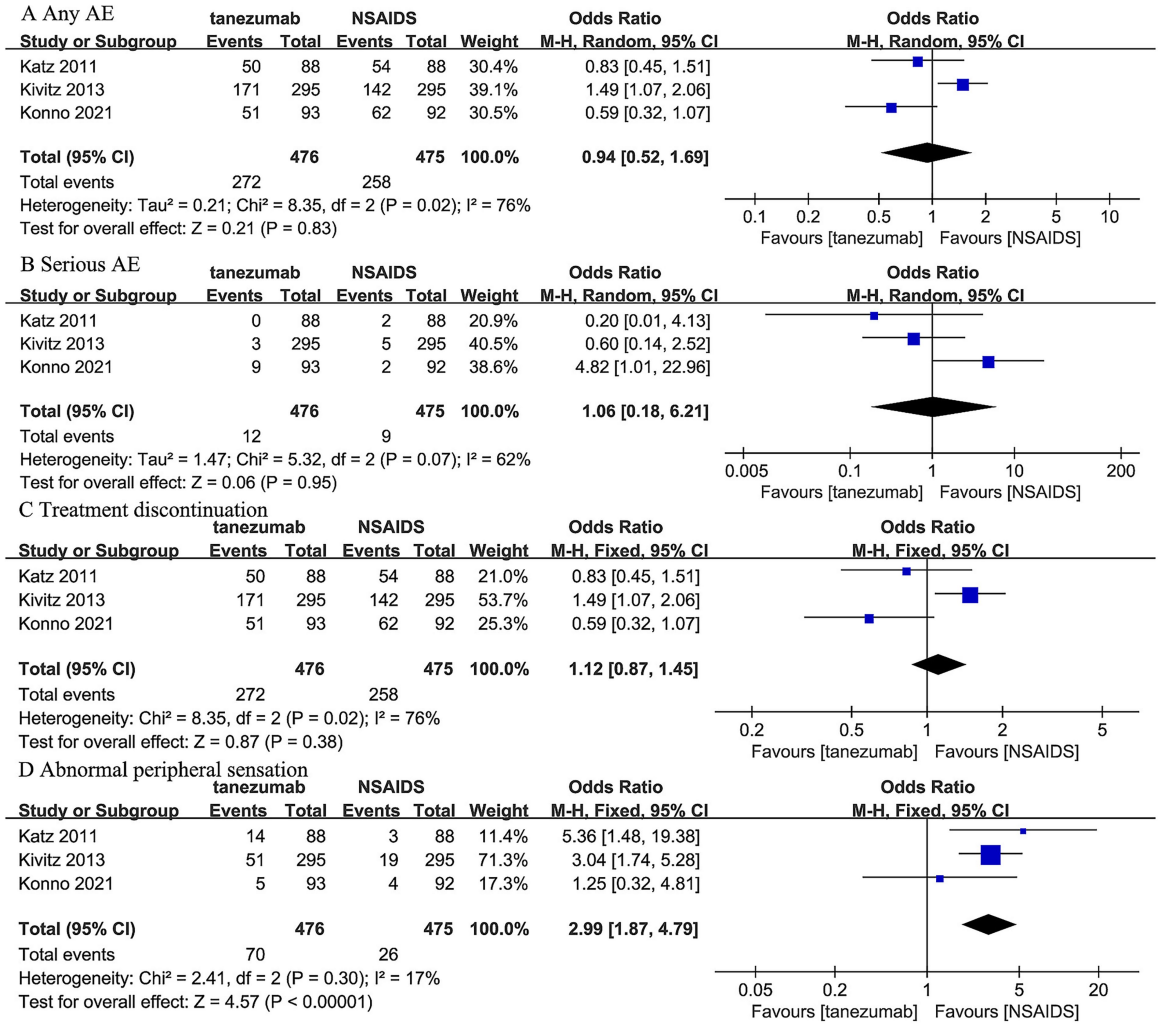
Meta-analysis of 10 mg tanezumab versus NSAIDs in **(A)** any AE, **(B)** serious AE, **(C)** treatment discontinuation due to AE, **(D)** abnormal peripheral sensation.

Three trials (44–46) reported that 10 mg tanezumab and NSAIDs exhibited similar results in any AE (OR, 0.94; 95% CI, 0.52 to 1.69; *I^2^* = 76%; *p* = 0.83), serious AE (OR, 1.06; 95% CI, 0.18 to 6.21; *I ^2^* = 62%; *p* = 0.95), and treatment discontinuation due to AE (OR, 1.12; 95% CI, 0.87 to 1.45; *I^2^* = 76%; *p* = 0.38). However, 10 mg tanezumab showed a significantly higher rate of abnormal peripheral sensation than NSAIDs (OR, 2.99; 95% CI, 1.87 to 4.79; *I^2^* = 17%; *p* < 0.001) (Figure 7).

Individual study provided further detail on specific safety concerns regarding joint safety. The Katz et al. (46) trial and Kivitz et al. (45) trial reported no joint safety event. However, the Konno et al. (44) trial, featuring an 80-week follow-up, observed rare but significant joint safety events. This included one patient in the 5 mg tanezumab group with RPOA type 1 in both knees, and two patients in the 10 mg tanezumab group with a subchondral insufficiency fracture of the left knee and RPOA type 2 of the left hip, respectively. The RPOA type 2 case notably led to the only total joint replacement reported across these three included studies. These specific RPOA events were identified in peripheral joints (knees and hips) with pre-existing possible-to-mild (Kellgren–Lawrence grade 1–2) radiographic evidence of OA at screening. However, similar joint destruction progression in the lumbar spine was not reported in these included trials.

### 3.6 Response rates

**Figure 8:**
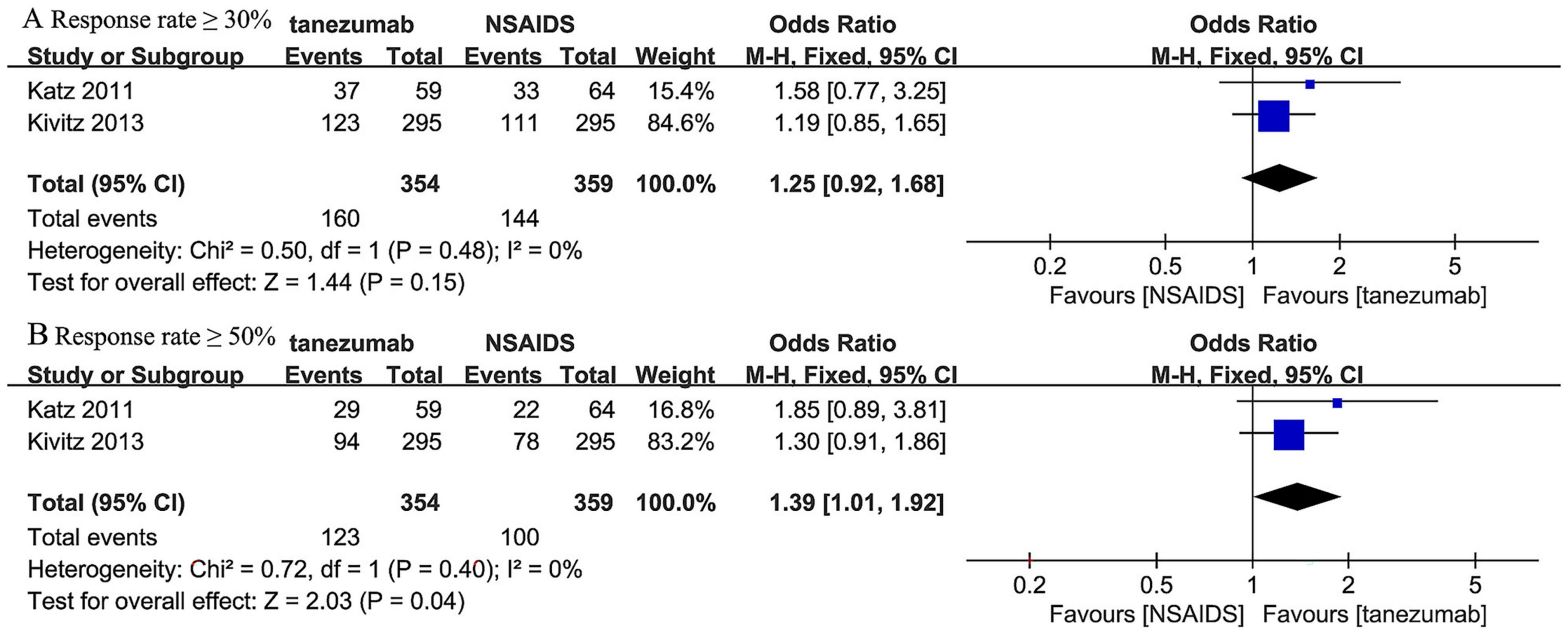
Meta-analysis of 10 mg tanezumab versus NSAIDs in **(A)** the incidence of response rates ≥ 30%, **(B)** the incidence of response rates ≥ 50% at over 12-week follow-up.

Three trials (45, 46) reported response rates at over 12-week follow-up (Figure 8). The 10 mg tanezumab group (160/354, 45.20%) had a slightly higher incidence of response rates ≥ 30% compared to the NSAIDs group (144/359, 40.11%), but the difference was not statistically significant (OR, 1.25; 95% CI, 0.92 to 1.68; *I^2^* = 0%; *p* = 0.15). Regarding the incidence of response rates ≥ 50%, the 10 mg tanezumab group (123/354, 34.75%) had a higher incidence than the NSAIDs group (100/359, 23.86%), and the difference was statistically significant (OR, 1.39; 95% CI, 1.01 to 1.92; *I^2^* = 0%; *p* = 0.04) (Figure 8).

## 4 Discussion

This present systematic review and meta-analysis comprehensively evaluated the efficacy and safety of tanezumab versus NSAIDs in the treatment of CLBP. In terms of pain reduction, tanezumab at the 10 mg dose, demonstrated a significantly greater reduction in LBPI scores at multiple time points, including the 1-week, 4-week, 8-week, and 12-week follow-ups, compared to NSAIDs. This suggests that tanezumab may offer superior short-term pain relief for individuals with CLBP. However, the 5 mg dose of tanezumab did not consistently show a better reduction in pain intensity than NSAIDs, with the exception of a slight but statistically significant improvement at the 8-week timepoint. The functional improvement, as measured by the RMDQ scores, was also significantly greater in the 10 mg tanezumab group than the NSAIDs group at the 2-week and 16-week follow-ups. The 5 mg dose, however, did not exhibit a significant difference in functional improvement compared to NSAIDs. In the safety profile, both the 5 mg and 10 mg doses of tanezumab showed similar rates of AEs to NSAIDs, with no significant differences in any AEs, serious AEs, or treatment discontinuation due to AEs. However, the 10 mg tanezumab group had a significantly higher rate of abnormal peripheral sensation than the NSAIDs group, a known concern with tanezumab treatment. The response rate analysis revealed that the 10 mg tanezumab group had a slightly higher but not statistically better incidence of response rate ≥ 30% and a statistically higher incidence of response rate ≥ 50% compared to the NSAIDs group, suggesting a greater proportion of patients achieving meaningful pain relief and functional improvement with tanezumab.

The findings reveal that tanezumab, particularly at a 10 mg dose, demonstrated significant superiority over NSAIDs in reducing low back pain intensity and enhancing physical function. These benefits were observed across various time points, indicating a sustained therapeutic effect. However, the 10 mg dose also presented a notable safety concern, with a significantly higher rate of peripheral sensation abnormalities compared to NSAIDs. This adverse effect may impact patient comfort and willingness to continue treatment, underscoring the importance of a careful risk–benefit assessment for each patient. On the other hand, the 5 mg dose of tanezumab did not consistently outperform NSAIDs in reducing pain intensity or improving functional outcomes. While it showed a statistically significant improvement at the 8-week mark, the overall results were less pronounced than those observed with the 10 mg dose. Notably, the 5 mg dose did not exhibit a significantly different AEs profile compared to NSAIDs, suggesting a more favorable safety outcome at this dosage. The inconsistency in the efficacy of 10 mg tanezumab at early time points warrants discussion. A significant reduction in LBPI was evident at 1 week across two trials, but this effect diminished at 2 weeks despite the addition of a third study, despite consistent efficacy at later time points (4, 8, 12, and 16 weeks). This discrepancy likely arises from the Katz et al. trial (46), which reported an opposing trend at 2 weeks compared to the other two trials (44, 45). In meta-analyses with few studies, a single contrasting result can skew the pooled estimate, particularly given the high heterogeneity. The reversal may reflect the potential placebo effect, common in early pain trial (47), the delayed pharmacokinetic action of tanezumab as an NGF antagonist (37, 46), and the limited number of studies, leading to unstable conclusions.

Recent systematic reviews and meta-analyses on anti-NGF antibodies for CLBP consistently report modest to moderate pain reduction and functional improvement compared to placebo, with dose-dependent effects. AE rates are generally comparable to placebo, though RPOA remains a notable concern (21, 34–37). Our meta-analysis, the first to compare tanezumab (5 mg and 10 mg) directly with NSAIDs in CLBP, aligns with these findings, showing that tanezumab 10 mg significantly reduces pain and improves function compared to NSAIDs, with higher ≥50% response rates, while the 5 mg dose offers no clear advantage. Nonetheless, these favorable findings are restricted by concerns regarding the drug’s safety profile, particularly at higher doses. In our study, we found that 5 mg tanezumab had similar safety profile compared to NSAIDs. As for 10 mg tanezumab, only a higher rate of abnormal peripheral sensation than NSAIDs was observed. Evidence indicates that the neurological changes are reversible, mild, and self-limiting, and there is no evidence suggesting that preexisting neurological conditions worsen with therapy (26, 37).

The development of tanezumab was significantly impacted by concerns regarding RPOA identified in patients with OA. Multiple pooled analyses (31–33) of Phase 3 OA trials have consistently revealed that adjudicated CJSEs, predominantly RPOA, occurred more frequently in tanezumab-treated groups compared to placebo or NSAIDs, exhibiting a dose-dependent risk. For instance, a secondary analysis by Miki et al. (33) showed CJSEs exclusively in tanezumab-treated patients at rates of 3.6% (2.5 mg) and 6.5% (5 mg), with no events in NSAID or placebo groups. Similarly, Berenbaum et al. (31) reported CJSE rates of 1.9% (2.5 mg) and 3.2% (5 mg) for tanezumab versus 0% for placebo. Furthermore, another pooled analysis by Carrino et al. (32) showed CJSEs in 3.2% of patients receiving tanezumab 2.5 mg and 6.2% on 5 mg tanezumab, compared to 1.5% in the NSAID group and 0% in the placebo group. Such severe joint-related adverse events ultimately led to the eventual discontinuation of tanezumab’s development for OA.

In this systematic review with meta-analysis, joint-related complications were specifically scrutinized. Among our three included RCTs, only one 80-week follow-up trial (44) reported specific joint safety events in the tanezumab group, with none in the NSAID group. This trial detailed one RPOA type 1 case (5 mg, both knees), one subchondral insufficiency fracture (10 mg, left knee), and one RPOA type 2 case (10 mg, left hip). This RPOA type 2 case notably led to the only total joint replacement reported across these three included studies of CLBP. While these peripheral joint complications were observed, RPOA or similar joint destruction in the lumbar spine was not reported in any included CLBP trial. Compared to the incidence of joint complications in OA trials (1.9 to 3.6% for 2.5 mg and 3.2 to 6.5% for 5 mg tanezumab), the very rare incidence of RPOA in our study (0.31% for 5 mg, 0.42% for 10 mg) precluded a meta-analysis on RPOA in CLBP trials. However, the distinct patterns of RPOA observed suggest a potential difference in risk profile between OA and CLBP populations. The RPOA case occurred in a patient with pre-existing mild osteoarthritis in the affected joint, suggesting pre-existing joint pathology as a significant risk factor, aligning with broader OA program observations (44). Thus, given the primary impact of PROA on peripheral joints (knees and hips) in OA trials and its rarity in CLBP trials (linked to pre-existing OA), the overall risk–benefit profile of tanezumab for CLBP appears more acceptable than for OA. This notable difference might position CLBP as a potentially safer indication for tanezumab, warranting further investigation into patient selection and risk stratification.

The cost-effectiveness of tanezumab for CLBP is a critical consideration. Total costs encompass the drug, administration, and monitoring expenses. The annual cost of the drug for the 5 mg dose ranges from $6,000 to $15,000, while the 10 mg dose spans from $12,000 to $24,000. Administration costs are higher for intravenous (IV) infusions compared to self-administered subcutaneous (SC) injections, with monitoring costs also varying: SC injections incur approximately $442 annually, whereas IV infusions cost around $250 (48). To assess cost-effectiveness, the Incremental Cost-Effectiveness Ratio (ICER) is calculated by comparing the additional costs of tanezumab with the additional quality-adjusted life years (QALYs) gained relative to NSAIDs (49). Based on cost-effectiveness models presented in existing literature (48), the ICER for the 5 mg dose ranges from $20,000 to $51,111 per QALY, and for the 10 mg dose, it ranges from $42,222 to $84,444 per QALY. It is typically considered cost-effective if the ICER falls below $50,000 per QALY, which suggests that tanezumab remains cost-effective at lower price points ($6,000–$12,000 annually) but becomes less cost-effective as prices exceed $15,000 annually (49). Although tanezumab offers superior pain relief and functional improvement, especially at the 10 mg dose, along with the advantage of less frequent dosing compared to daily NSAIDs, its high cost remains a major limitation.

This present study underscores the potential of tanezumab for managing CLBP, particularly at the 10 mg dose. However, its integration into standard clinical practice requires careful consideration. Clinical development remains suspended due to safety concerns from larger OA trials, resulting in no established guidelines. Nonetheless, the findings offer insights into its potential role. Clinicians should target patients with moderate to severe CLBP unresponsive to NSAIDs or with contraindications. This would require screening for pre-existing OA to mitigate the risks of rare RPOA, alongside vigilant monitoring for peripheral sensation and other joint-related adverse events. The 5 mg dose, offering a safety profile comparable to NSAIDs, may suit milder cases or higher-risk patients but lacks significant efficacy superiority. Administration via subcutaneous injections every 8 weeks could enhance adherence over daily NSAID use, though this demands adequate injection infrastructure and patient education. Cost-effectiveness poses a significant challenge, and it remains cost-effective only when prices stay below $15,000 annually. Thus, clinical adoption depends on balancing its pain relief and functional benefits against safety and economic constraints, requiring strict monitoring and alignment with local policies, pending further long-term data. There are some limitations of the study. While we included all high-quality trials on this topic, the limited number of available RCTs may have constrained the breadth of our analysis and the generalizability of our findings. Second, unavoidable heterogeneity existed among the included studies regarding patient demographics, intervention protocols, and follow-up periods, further compounded by the limited number of studies, resulting in significant heterogeneity of outcomes. While univariate and multivariate meta-regression are necessary to thoroughly explore sources of such heterogeneity, these analyses were precluded by the constrained number of included studies. Third, the relatively short duration of follow-up in some studies may have prevented a comprehensive assessment of long-term efficacy and safety outcomes. Additionally, only one included study reported the incidence of RPOA, precluding a meta-analysis on this critical concern. Last, variations in the primary etiology of CLBP among different trials made it challenging to determine the most suitable use for tanezumab. Further research is warranted to elucidate the effectiveness of tanezumab in treating different causative pathologies of CLBP.

## 5 Conclusion

Tanezumab at 10 mg demonstrates better pain relief and functional improvement for CLBP compared to NSAIDs but comes with an increased risk of mild peripheral sensation abnormalities. In contrast, the 5 mg dose shows a comparable safety profile to NSAIDs but does not provide significant therapeutic advantages. While joint safety events significantly impacted development of tanezumab for OA, their rare occurrence in peripheral joints with pre-existing OA and absence in the lumbar spine within CLBP trials suggests its risk–benefit profile appears more acceptable in CLBP than in OA. Clinicians must therefore balance benefits, risks, and cost-effectiveness when considering tanezumab for CLBP. More high-quality CLBP research is urgently needed for refined treatment strategies.

## Data Availability

The original contributions presented in the study are included in the article/Supplementary material, further inquiries can be directed to the corresponding author.
