# Supplementary material for: Comparison of tanezumab and non-steroidal anti-inflammatory drugs in efficacy and safety for chronic low back pain: a systematic review and meta-analysis of randomized controlled trials
Source: Front Neurol. 2025 Sep 1;16:1623280. doi: 10.3389/fneur.2025.1623280 (PMC12434963; doi:10.3389/fneur.2025.1623280)
Supplement: Supplementary file 1 [file Table_1.DOCX]

**Supplementary Table 1:** Study selection.

| **No.** | **Study title** | **First author** | **Year** | **Included/excluded** | **Reason for exclusion (if applicable)** | **Screening process** | **Date** |
| --- | --- | --- | --- | --- | --- | --- | --- |
| 1 | Efficacy and safety of tanezumab in the treatment of chronic low back pain | Katz, N | 2011 | Included | NA | Full-text screened by Y.D. and P.L., consensus reached on inclusion. | 2025.3.15 |
| 2 | Efficacy and safety of tanezumab versus naproxen in the treatment of chronic low back pain | Kivitz, A. J. | 2013 | Included | NA | Full-text screened by Y.D. and P.L., consensus reached on inclusion. | 2025.3.15 |
| 3 | Nerve growth factor: an update on the science and therapy | Seidel, M. F. | 2013 | Excluded | This is a review. | Abstract screened by Y.D. and P.L., consensus reached on exclusion. | 2025.3.15 |
| 4 | Long-term safety and effectiveness of tanezumab as treatment for chronic low back pain | Gimbel, J. S. | 2014 | Excluded | No NSAIDS group. Patients were randomized to tanezumab 10 mg (n = 321) or 20 mg (n = 527) administered at 8-week intervals via 3 intravenous injections fol lowed by 4 subcutaneous injections. | Full-text screened by Y.D. and P.L., consensus reached on exclusion. | 2025.3.15 |
| 5 | Anti-nerve growth factor in the treatment of low back pain and radiculopathy: a systematic review and a meta-analysis | Leite, V. F. | 2014 | Excluded | This is a systematic review and meta-analysis. | Abstract screened by Y.D. and P.L., consensus reached on exclusion. | 2025.3.15 |
| 6 | Efficacy, Safety, and Tolerability of Fulranumab as an Adjunctive Therapy in Patients With Inadequately Controlled, Moderate-to-Severe Chronic Low Back Pain: A Randomized, Double-blind, Placebo-controlled, Dose-ranging, Dose-loading Phase II Study | Sanga, P. | 2016 | Excluded | No tanezumab or NSAIDS group. This is a randomized, double-blind, placebo-controlled, dose-ranging, dose-loading Phase II study comparing Fulranumab to placebo. | Abstract screened by Y.D. and P.L., consensus reached on exclusion. | 2025.3.15 |
| 7 | Nerve Growth Factor Antagonists: Is the Future of Monoclonal Antibodies Becoming Clearer? | Bannwarth, B. | 2017 | Excluded | This is a review. | Abstract screened by Y.D. and P.L., consensus reached on exclusion. | 2025.3.15 |
| 8 | Tanezumab in the treatment of chronic musculoskeletal conditions | Jayabalan, P. | 2017 | Excluded | This is a review. | Abstract screened by Y.D. and P.L., consensus reached on exclusion. | 2025.3.15 |
| 9 | Tanezumab: Therapy targeting nerve growth factor in pain pathogenesis | Patel, M. K. | 2018 | Excluded | This is a review. | Abstract screened by Y.D. and P.L., consensus reached on exclusion. | 2025.3.15 |
| 10 | Tanezumab: a selective humanized mAb for chronic lower back pain | Webb, M. P. | 2018 | Excluded | This is a review. | Abstract screened by Y.D. and P.L., consensus reached on exclusion. | 2025.3.15 |
| 11 | Tanezumab for chronic low back pain: a randomized, double-blind, placebo- and active-controlled, phase 3 study of efficacy and safety | Markman, J. D. | 2020 | Excluded | No NSAIDS group. This is a randomized, double-blind, placebo- and active-controlled, phase 3 study. Patients received placebo, subcutaneous tanezumab (5 or 10 mg every 8 weeks), or oral tramadol prolonged-release (100-300 mg/day). | Full-text screened by Y.D. and P.L., consensus reached on exclusion. | 2025.3.15 |
| 12 | Anti-nerve growth factor antibodies for the treatment of low back pain | Patel, F. | 2020 | Excluded | This is a review. | Abstract screened by Y.D. and P.L., consensus reached on exclusion. | 2025.3.15 |
| 13 | The Efficacy of Nerve Growth Factor Antibody for the Treatment of Osteoarthritis Pain and Chronic Low-Back Pain: A Meta-Analysis | Yang, S. | 2020 | Excluded | This is a meta-analysis. | Abstract screened by Y.D. and P.L., consensus reached on exclusion. | 2025.3.15 |
| 14 | Is Targeting Nerve Growth Factor Antagonist a New Option for Pharmacologic Treatment of Low Back Pain? A Supplemental Network Meta-Analysis of the American College of Physicians Guidelines | Cao, Z. | 2021 | Excluded | This is a review. | Abstract screened by Y.D. and P.L., consensus reached on exclusion. | 2025.3.15 |
| 15 | Efficacy and safety of fasinumab in patients with chronic low back pain: a phase II/III randomised clinical trial | Dakin, P. | 2021 | Excluded | No tanezumab or NSAIDS group. This is a randomized, double-blind, placebo-controlled, Phase II/III study comparing Fulranumab to placebo. | Abstract screened by Y.D. and P.L., consensus reached on exclusion. | 2025.3.15 |
| 16 | The evolution of nerve growth factor inhibition in clinical medicine | Wise, B. L. | 2021 | Excluded | This is a review. | Abstract screened by Y.D. and P.L., consensus reached on exclusion. | 2025.3.15 |
| 17 | Tanezumab for chronic low back pain: a long-term, randomized, celecoxib-controlled Japanese Phase III safety study | Konno, S. I. | 2022 | Included | NA | Full-text screened by Y.D. and P.L., consensus reached on inclusion. | 2025.3.15 |
| 18 | Clinical Meaningfulness of Response to Tanezumab in Patients with Chronic Low Back Pain: Analysis From a 56-Week, Randomized, Placebo- and Tramadol-Controlled, Phase 3 Trial | Markman, J. D. | 2022 | Excluded | No NSAIDS group. This is a randomized, Placebo- and Tramadol-Controlled, Phase 3 Trial. Patients received placebo (up to week 16; n = 406), subcutaneously administered (SC) tanezumab 5 mg (every 8 weeks; n = 407), SC tanezumab 10 mg (every 8 weeks; n = 407), or orally administered tramadol prolonged-release (100-300 mg/day; n = 605) for 56 weeks. | Full-text screened by Y.D. and P.L., consensus reached on exclusion. | 2025.3.15 |
| 19 | Population pharmacokinetics of tanezumab following intravenous or subcutaneous administration to patients with osteoarthritis or chronic low back pain | Shoji, S. | 2022 | Excluded | This is a pharmacokinetics study. | Abstract screened by Y.D. and P.L., consensus reached on exclusion. | 2025.3.15 |
| 20 | Different Dosage Regimens of Tanezumab for the Treatment of Chronic Low Back Pain: A Meta-analysis of Randomized Controlled Trials | Lian, J. | 2023 | Excluded | This is a systematic review and meta-analysis. | Abstract screened by Y.D. and P.L., consensus reached on exclusion. | 2025.3.15 |
| 21 | Various Doses of Tanezumab in the Management of Chronic Low Back Pain (CLBP): A Pooled Analysis of 4,514 Patients | Tahir, S. | 2023 | Excluded | This is a pooled analysis. | Abstract screened by Y.D. and P.L., consensus reached on exclusion. | 2025.3.15 |

Note: These are the studies after deduplication from the literature search.
