# Supplementary material for: Comparison of tanezumab and non-steroidal anti-inflammatory drugs in efficacy and safety for chronic low back pain: a systematic review and meta-analysis of randomized controlled trials
Source: Front Neurol. 2025 Sep 1;16:1623280. doi: 10.3389/fneur.2025.1623280 (PMC12434963; doi:10.3389/fneur.2025.1623280)
Supplement: Supplementary file 2 [file Table_2.DOCX]

**Supplementary Table 2:** Extraction table.

1. Efficacy and safety of tanezumab in the treatment of chronic low back pain (Katz et al., 2011)

| Extractors: D.Y., P.L. | EXtraction date: 2025.3.20 | | | | | | |
| --- | --- | --- | --- | --- | --- | --- | --- |
| Author | Katz et al. | | | | | | |
| Title | Efficacy and safety of tanezumab in the treatment of chronic low back pain | | | | | | |
| Year of publication | 2011 | | | | | | |
| Country | USA | | | | | | |
| NCT | NCT00924664 | | | | | | |
| sTUDY DESIGN | Multicenter RCT | | | | | | |
| Trial PHASE | II | | | | | | |
| Level of evidence | I | | | | | | |
| sTUDY arms | - Single i.v. infusion of tanezumab 200 lg/kg with matching oral placebo for naproxen (tanezumab group) (n = 88); - Single i.v. infusion of tanezumab placebo (vehicle) with naproxen 1000 mg/d (naproxen group) (n = 88); - Single i.v. infusion of tanezumab placebo with matching oral placebo for naproxen (placebo group) (n = 41). | | | | | | |
| Participants inclusion criteria | - 1. Age >18 years and BMI ≤39 kg/m2; - 2. CLBP ≥3 months requiring regular use of analgesic medication (>4 days/week for the past month); - 3. Primary location of low back pain is between the 12th thoracic vertebra and the lower gluteal folds, with or without radiation into the posterior thigh, classified as category 1 or 2 according to the classification of the Quebec Task Force in Spinal Disorders; - 4. Must have a score of ≥4 for LBPI while on current treatment at screening and completes at least 4 daily pain diaries during the 5 d before randomization, with an average LBPI score of ≥4. | | | | | | |
| Participants exclusion criteria | - 1. Lumbosacral radiculopathy within the past 2 years; - 2. Spinal stenosis associated with neurological impairment or neurogenic claudication; - 3. Back pain due to visceral disorder; - 4. Patients with history, diagnosis, or signs and symptoms of clinically significant neurological disease, recent major trauma, osteoporotic compression fracture, or surgical intervention for the treatment of LBP; - 5. Pregnancy or lactation; - 6. Rheumatoid arthritis; - 7. Seronegative spondyloarthropathy; - 8. Paget disease of the spine, pelvis, or femur; fibromyalgia; - 9. Tumors or infections of the spinal cord; - 10. Cancer within the last 2 years, other than cutaneous basal cell or squamous cell carcinoma resolved by excision; - 11. Allergic or anaphylactic reaction to a therapeutic or diagnostic monoclonal antibody or IgG fusion protein; 12. Intolerance to acetaminophen or any of its excipients; - 13. Hypersensitivity to NSAIDs or any condition that might have precluded the use of an NSAID; - 14. Clinically significant cardiovascular, chronic viral, or neurological disease; - 15.Psychiatric disorders. | | | | | | |
| fOLLOW-UP | Patients had study visits on Days 8, 15, 29, 43, 57, and 85 (ie, Weeks 1, 2, 4, 6, 8, and 12), during which safety and efficacy assessments, routine laboratory tests, and blood samples were obtained. A follow-up visit on Day 113 (ie, Week 16) allowed assessment of the safety and tolerability of tanezumab. | | | | | | |
| cONFIRMATION OF eligiblILITY | D.Y. and P.L. independently reviewed the full texts to confirm their eligibility for inclusion in this review, reaching consensus on the final selections. | | | | | | |
| data source and MISSING DATA | All baseline data in this table are original data extracted directly from the source articles. The data used for analysis were extracted from the figures and tables in the original articles.For those without reported standard deviations (SDs), the SDs were estimated using the formula: SD ≈ Range/4. | | | | | | |
| Participant characteristics* (*: Only the arms that were included in meta-analyses are represented in this table.) | **Age, mean ± SD, y** | - 49.5 ± 14.7 (tanezumab group); - 52.1 ± 14.8 (naproxen group). | | | | | |
|  | **Gender, n (%)** | - Male: 35 (39.8), female: 53 (60.2) (tanezumab group); - Male: 46 (52.3), female: 42 (47.7) (naproxen group). | | | | | |
|  | **Race, n (%)** | - White: 81 (92.0) black: 1 (1.1) asian: 2 (2.3) other: 4 (4.5) (tanezumab group); - White: 82 (93.2) black: 5 (5.7) asian: 0 other: 1 (1.1) (naproxen group). | | | | | |
|  | **BMI, mean ± SD, kg/m^2^** | - 28.8 ± 4.8 (tanezumab group); - 28.6 ± 4.8 (naproxen group). | | | | | |
|  | **Duration of CLBP, mean (range), y** | - 10.0 (0.3–48.3) (tanezumab group); - 13.0 (0.4–52.9) (naproxen group). | | | | | |
|  | **Primary etiology, n (%)** | - Disc disease:30 (34.1) degenerative joint disease/OA: 33 (37.5) injury/muscular strain: 21 (23.9) other: 4 (4.5) (tanezumab group); - Disc disease:21 (23.9) degenerative joint disease/OA: 37 (42.0) injury/muscular strain: 20 (22.7) other: 10 (11.4) (naproxen group). | | | | | |
|  | **LBPI, mean ± SD** | - 6.5 ± 1.4 (tanezumab group); - 6.7 ± 1.4 (naproxen group). | | | | | |
|  | **RMDQ, mean ± SD** | - 12.3 ± 4.6 (tanezumab group); - 12.4 ± 4.8 (naproxen group). | | | | | |
| DATA for meta-analysis* (*: Only the arms that were included in meta-analyses are represented in this table.) | Continuous variables | Group | Sample size | Mean | Confidence Interval (Lower) | Confidence Interval (Upper) | Standard deviation |
|  | Δ LBPI at 1-week: 10 mg tanezumab versus NSAIDs | Tanezumab | 59 | -2.42 | -2.63 | -2.04 | 0.15 |
|  |  | NSAIDs | 64 | -2.14 | -2.36 | -1.94 | 0.11 |
|  | Δ LBPI at 2-week: 10 mg tanezumab versus NSAIDs | Tanezumab | 59 | -2.23 | -2.44 | -2 | 0.11 |
|  |  | NSAIDs | 64 | -2.39 | -2.59 | -2.16 | 0.11 |
|  | Δ LBPI at 4-week: 10 mg tanezumab versus NSAIDs | Tanezumab | 59 | -3.18 | -3.4 | -2.43 | 0.11 |
|  |  | NSAIDs | 64 | -2.43 | -2.63 | -2.23 | 0.1 |
|  | Δ LBPI at 8-week: 10 mg tanezumab versus NSAIDs | Tanezumab | 59 | -3.37 | -3.59 | -3.15 | 0.11 |
|  |  | NSAIDs | 64 | -2.54 | -2.76 | -2.33 | 0.1 |
|  | Δ LBPI at 12-week: 10 mg tanezumab versus NSAIDs | Tanezumab | 59 | -3.03 | -3.51 | -3.27 | 0.06 |
|  |  | NSAIDs | 64 | -2.91 | -2.68 | -2.48 | 0.05 |
|  | Δ RMQD at 2-week: 10 mg tanezumab versus NSAIDs | Tanezumab | 59 | -5.43 | -6.01 | -4.84 | 0.29 |
|  |  | NSAIDs | 64 | -3.96 | -4.54 | -3.38 | 0.29 |
|  | Δ RMQD at 8-week: 10 mg tanezumab versus NSAIDs | Tanezumab | 59 | -7.45 | -8.09 | -6.8 | 0.32 |
|  |  | NSAIDs | 64 | -4.9 | -5.52 | -4.29 | 0.31 |
|  | Dichotomous variables | Group | | Events | | Total | |
|  | Any AE: 10 mg tanezumab versus NSAIDs | Tanezumab | | 50 | | 88 | |
|  |  | NSAIDs | | 54 | | 88 | |
|  | Serious AE: 10 mg tanezumab versus NSAIDs | Tanezumab | | 0 | | 88 | |
|  |  | NSAIDs | | 2 | | 88 | |
|  | Treatment discontiuation: 10 mg tanezumab versus NSAIDs | Tanezumab | | 50 | | 88 | |
|  |  | NSAIDs | | 54 | | 88 | |
|  | Abonormal peripheral sensation: 10 mg tanezumab versus NSAIDs | Tanezumab | | 14 | | 88 | |
|  |  | NSAIDs | | 3 | | 88 | |
|  | Response rate ≥30%: 10 mg tanezumab versus NSAIDs | Tanezumab | | 37 | | 59 | |
|  |  | NSAIDs | | 33 | | 64 | |
|  | Response rate ≥50%: 10 mg tanezumab versus NSAIDs | Tanezumab | | 29 | | 59 | |
|  |  | NSAIDs | | 22 | | 64 | |

2. Efficacy and safety of tanezumab versus naproxen in the treatment of chronic low back pain (Kivitz et al., 2013)

| Extractors: D.Y., P.L. | EXtraction date: 2025.3.20 | | | | | | |
| --- | --- | --- | --- | --- | --- | --- | --- |
| Author | Kivitz et al. | | | | | | |
| Title | Efficacy and safety of tanezumab versus naproxen in the treatment of chronic low back pain | | | | | | |
| Year of publication | 20133 | | | | | | |
| Country | USA | | | | | | |
| NCT | NCT00876187 | | | | | | |
| sTUDY DESIGN | Multicenter RCT | | | | | | |
| Trial PHASE | II | | | | | | |
| Level of evidence | I | | | | | | |
| sTUDY arms | - IV Tanezumab 5 mg at baseline and week 8 (n = 232); - IV Tanezumab 10 mg at baseline and week 8 (n = 295); - IV Tanezumab 20 mg at baseline and week 8 (n = 295); - Placebo (n = 295); - Naproxen 500 mg b.i.d. daily (n = 295). | | | | | | |
| Participants inclusion criteria | - 1. CLBP ≥3 months requiring regular use of analgesic medication (>4 days/week for the past month) including immediate-release opioids (in which the average daily opioid dose [for a 7-day period] did not exceed a morphine equivalent dose of 30 mg/day) but excluding acetaminophen, gabapentin, or pregabalin as the sole analgesics used for CLBP; - 2. Primary location of low back pain between the 12th thoracic vertebra and the lower gluteal folds, with or without radiation into the posterior thigh (Quebec Task Force on Spinal Disorders category 1 or 2); - 3. Average LBPI score of ≥4 while receiving current treatment; - 4. Patient’s Global Assessment of Low Back Pain of fair, poor, or very poor. | | | | | | |
| Participants exclusion criteria | - 1. Lumbosacral radiculopathy within the past 2 years, vertebral fracture, major trauma, or back surgery in the past 6 months; - 2. Significant cardiac, neurological, or other pain, or psychological conditions; - 3. Known history of rheumatoid arthritis, seronegative spondyloarthropathy; - 4. Paget disease of the spine, pelvis, or femur; - 5. Fibromyalgia; tumors or infections of the spinal cord; - 6. Any condition that might preclude NSAID use; - 7. Patients also were excluded if extended-release opioids or long-acting opioids such as oxycodone controlled-release, oxymorphone extended-release, hydromorphone, transdermal fentanyl, or methadone had been used within 3 months of screening. | | | | | | |
| fOLLOW-UP | Study visits were conducted at screening, baseline (randomization; day 0), and weeks 2, 4, 8, 12, and 16. | | | | | | |
| cONFIRMATION OF eligiblILITY | D.Y. and P.L. independently reviewed the full texts to confirm their eligibility for inclusion in this review, reaching consensus on the final selections. | | | | | | |
| data source and MISSING DATA | All baseline data in this table are original data extracted directly from the source articles. The data used for analysis were extracted from the figures and tables in the original articles.For those without reported standard deviations (SDs), the SDs were estimated using the formula: SD ≈ Range/4. | | | | | | |
| Participant characteristics* (*: Only the arms that were included in meta-analyses are represented in this table.) | **Age, mean ± SD, y** | - 51.5 ± 11.7 (5 mg tanezumab group); - 52 ± 11.0 (10 mg tanezumab group); - 52.6 ± 11.5 (naproxen group). | | | | | |
|  | **Gender, n (%)** | - Male: 117 (50.4), female: 115 (49.6) (5 mg tanezumab group); - Male: 138 (46.8), female: 157 (53.2) (10 mg tanezumab group); - Male: 143 (48.5), female: 152 (51.5) (naproxen group). | | | | | |
|  | **Race, n (%)** | - White: 187 (80.6) black: 32 (13.8) asian: 4 (1.7) other: 9 (3.9) (5 mg tanezumab group); - White: 238 (80.7) black: 43 (14.6) asian: 5 (1.7) other: 9 (3.1) (10 mg tanezumab group); - White: 224 (75.9) black: 57 (19.3) asian: 3 (1.0) other: 11 (3.7) (naproxen group). | | | | | |
|  | **BMI, mean ± SD, kg/m^2^** | - 29.2 ± 4.9 (5 mg tanezumab group); - 29.3 ± 4.9 (10 mg tanezumab group); - 30.3 ± 5.0 (naproxen group). | | | | | |
|  | **Duration of CLBP, mean (range), y** | - 10.9 (0.3–67.7) (5 mg tanezumab group); - 11.2 (0.4–55.9) (10 mg tanezumab group). - 11.2 (0.3–53.9) (naproxen group). | | | | | |
|  | **Primary etiology, n (%)** | - Disc disease:64 (27.6) degenerative joint disease/OA: 90 (38.8) injury/muscular strain: 73 (31.5) other: 5 (2.2) (5 mg tanezumab group); - Disc disease:82 (27.8) degenerative joint disease/OA: 98 (33.2) injury/muscular strain: 109 (36.9) other:6 (2.0) (10 mg tanezumab group); - Disc disease:69 (23.4) degenerative joint disease/OA: 125 (42.4) injury/muscular strain: 96 (32.5) other: 5 (1.7) (naproxen group). | | | | | |
|  | **LBPI, mean ± SD** | - 6.62 ± 1.4 (5 mg tanezumab group); - 6.57 ± 1.4 (10 mg tanezumab group); - 6.77 ± 1.4 (naproxen group). | | | | | |
|  | **RMDQ, mean ± SD** | - 12.24 ± 4.9 (5 mg tanezumab group); - 12.98 ± 5.1 (10 mg tanezumab group); - 12.86 ± 4.9 (naproxen group). | | | | | |
| DATA for meta-analysis* (*: Only the arms that were included in meta-analyses are represented in this table.) | Continuous variables | Group | Sample size | Mean | Confidence Interval (Lower) | Confidence Interval (Upper) | Standard deviation |
|  | Δ LBPI at 2-week: 5 mg tanezumab versus NSAIDs | Tanezumab | 232 | -1.4 | -1.66 | -1.53 | 0.03 |
|  |  | NSAIDs | 295 | -1.7 | -1.94 | -1.82 | 0.03 |
|  | Δ LBPI at 4-week: 5 mg tanezumab versus NSAIDs | Tanezumab | 232 | -1.98 | -2.13 | -1.86 | 0.07 |
|  |  | NSAIDs | 295 | -2.02 | -2.15 | -1.89 | 0.07 |
|  | Δ LBPI at 8-week: 5 mg tanezumab versus NSAIDs | Tanezumab | 232 | -1.87 | -2.02 | -1.72 | 0.08 |
|  |  | NSAIDs | 295 | -1.79 | -1.92 | -1.66 | 0.07 |
|  | Δ LBPI at 12-week: 5 mg tanezumab versus NSAIDs | Tanezumab | 232 | -1.96 | -2.12 | -1.8 | 0.08 |
|  |  | NSAIDs | 295 | -1.9 | -2.04 | -1.76 | 0.07 |
|  | Δ LBPI at 16-week: 5 mg tanezumab versus NSAIDs | Tanezumab | 232 | -1.58 | -1.74 | -1.41 | 0.08 |
|  |  | NSAIDs | 295 | -1.66 | -1.8 | -1.53 | 0.07 |
|  | Δ LBPI at 2-week: 10 mg tanezumab versus NSAIDs | Tanezumab | 232 | -1.88 | -2.01 | -1.76 | 0.06 |
|  |  | NSAIDs | 295 | -1.7 | -1.78 | -1.66 | 0.03 |
|  | Δ LBPI at 4-week: 10 mg tanezumab versus NSAIDs | Tanezumab | 232 | -2.54 | -2.66 | -2.4 | 0.07 |
|  |  | NSAIDs | 295 | -2.02 | -2.16 | -1.9 | 0.07 |
|  | Δ LBPI at 8-week: 10 mg tanezumab versus NSAIDs | Tanezumab | 232 | -2.29 | -2.42 | -2.16 | 0.77 |
|  |  | NSAIDs | 295 | -1.79 | -1.92 | -1.63 | 0.07 |
|  | Δ LBPI at 12-week: 10 mg tanezumab versus NSAIDs | Tanezumab | 232 | -2.3 | -2.44 | -2.16 | 0.07 |
|  |  | NSAIDs | 295 | -1.9 | -2.01 | -1.73 | 0.07 |
|  | Δ LBPI at 16-week: 10 mg tanezumab versus NSAIDs | Tanezumab | 232 | -2.07 | -2.21 | -1.92 | 0.07 |
|  |  | NSAIDs | 295 | -1.66 | -1.78 | -1.5 | 0.07 |
|  | Δ RMQD at 16-week: 10 mg tanezumab versus NSAIDs | Tanezumab | 232 | -2.37 | -3.08 | -2.02 | 0.29 |
|  |  | NSAIDs | 295 | -2.07 | -2.51 | -1.47 | 0.26 |
|  | Δ RMQD at 16-week: 5 mg tanezumab versus NSAIDs | Tanezumab | 232 | -2.37 | -3.08 | -2.02 | 0.29 |
|  |  | NSAIDs | 295 | -2.07 | -2.51 | -1.47 | 0.26 |
|  | Dichotomous variables | Group | | Events | | Total | |
|  | Any AE: 5 mg tanezumab versus NSAIDs | Tanezumab | | 141 | | 232 | |
|  |  | NSAIDs | | 142 | | 295 | |
|  | Serious AE: 5 mg tanezumab versus NSAIDs | Tanezumab | | 4 | | 232 | |
|  |  | NSAIDs | | 5 | | 295 | |
|  | Treatment discontiuation: 5 mg tanezumab versus NSAIDs | Tanezumab | | 11 | | 232 | |
|  |  | NSAIDs | | 10 | | 295 | |
|  | Abonormal peripheral sensation: 5 mg tanezumab versus NSAIDs | Tanezumab | | 25 | | 232 | |
|  |  | NSAIDs | | 19 | | 295 | |
|  | Any AE: 10 mg tanezumab versus NSAIDs | Tanezumab | | 171 | | 295 | |
|  |  | NSAIDs | | 142 | | 295 | |
|  | Serious AE: 10 mg tanezumab versus NSAIDs | Tanezumab | | 3 | | 295 | |
|  |  | NSAIDs | | 5 | | 295 | |
|  | Treatment discontiuation: 10 mg tanezumab versus NSAIDs | Tanezumab | | 171 | | 295 | |
|  |  | NSAIDs | | 142 | | 295 | |
|  | Abonormal peripheral sensation: 10 mg tanezumab versus NSAIDs | Tanezumab | | 51 | | 295 | |
|  |  | NSAIDs | | 19 | | 295 | |
|  | Response rate ≥30%: 10 mg tanezumab versus NSAIDs | Tanezumab | | 123 | | 295 | |
|  |  | NSAIDs | | 111 | | 295 | |
|  | Response rate ≥50%: 10 mg tanezumab versus NSAIDs | Tanezumab | | 94 | | 295 | |
|  |  | NSAIDs | | 78 | | 295 | |

3. Tanezumab for chronic low back pain: a long-term, randomized, celecoxib controlled Japanese Phase III safety study (Konno et al., 2022)

| Extractors: D.Y., P.L. | EXtraction date: 2025.3.20 | | | | | | |
| --- | --- | --- | --- | --- | --- | --- | --- |
| Author | Konno et al. | | | | | | |
| Title | Tanezumab for chronic low back pain: a long-term, randomized, celecoxib controlled Japanese Phase III safety study | | | | | | |
| Year of publication | 2022 | | | | | | |
| Country | Japan | | | | | | |
| NCT | NCT02725411 | | | | | | |
| sTUDY DESIGN | Multicenter RCT | | | | | | |
| Trial PHASE | III | | | | | | |
| Level of evidence | I | | | | | | |
| sTUDY arms | - SC tanezumab 5 mg + oral placebo (n = 92); - SC tanezumab 10 mg + oral placebo (n = 93);   Oral celecoxib + SC placebo (n = 92). | | | | | | |
| Participants inclusion criteria | - 1. Patients aged ≥18 years with CLBP (primary location between the 12th thoracic vertebra and lower gluteal folds, with or without radiation into the posterior thigh [category 1 or 2 per Quebec Task Force in Spinal Disorders]) of ≥3 months’ duration; - 2. LBPI score ≥5 at screening and baseline; - 3. Patient’s Global Assessment of Low Back Pain score of fair, poor, or very poor at baseline; - 4. Patients were required to be experiencing some benefit from, and ability to tolerate, a stable (≥5 days/week in the 30 days prior to baseline) regimen of oral NSAID therapy (celecoxib 100 mg twice daily, loxoprofen 120–180 mg/day, or meloxicam 5–15 mg/day) but still require additional pain relief at screening. | | | | | | |
| Participants exclusion criteria | - 1. A history of lumbosacral radiculopathy; - 2. Diagnosis of osteoarthritis of the knee or hip based on American College of Rheumatology combined clinical and radiographic criteria, KL-based radiographic evidence of hip (grade ≥2) or knee (grade ≥3) osteoarthritis; - 3. Radiographic evidence and symptoms of osteoarthritis of the shoulders. | | | | | | |
| fOLLOW-UP | The study included 56-week treatment and 24-week safety follow-up periods. Efficacy assessments were made at baseline and weeks 1, 2, 4, 8, 12, 16, 24, 32, 40, 48, 56 and 64. | | | | | | |
| cONFIRMATION OF eligiblILITY | D.Y. and P.L. independently reviewed the full texts to confirm their eligibility for inclusion in this review, reaching consensus on the final selections. | | | | | | |
| data source and MISSING DATA | All baseline data in this table are original data extracted directly from the source articles. The data used for analysis were extracted from the figures and tables in the original articles.For those without reported standard deviations (SDs), the SDs were estimated using the formula: SD ≈ Range/4. | | | | | | |
| Participant characteristics* (*: Only the arms that were included in meta-analyses are represented in this table.) | **Age, mean ± SD, y** | - 53.3 ± 14.25 (5 mg tanezumab group); - 52.3 ± 14.25 (10 mg tanezumab group); - 54.3 ± 15.5 (Celecoxib group). | | | | | |
|  | **Gender, n (%)** | - Male: 55 (59.8), female: 37 (40.2) (5 mg tanezumab group); - Male: 49 (52.7), female: 44 (47.3) (10 mg tanezumab group); - Male: 54 (58.7), female: 38 (41.3) (Celecoxib group). | | | | | |
|  | **Race, n (%)** | - White: 0 (0) black: 0 (0) asian: 92 (100) other: 0 (0) (5 mg tanezumab group); - White: 0 (0) black: 0 (0) asian: 93 (100) other: 0 (0) (10 mg tanezumab group); - White: 0 (0) black: 0 (0) asian: 92 (100) other: 0 (0) (Celecoxib group). | | | | | |
|  | **BMI, mean ± SD, kg/m^2^** | - 24.1 ± 3.9 (5 mg tanezumab group); - 23.9 ± 4.2 (10 mg tanezumab group); - 23.9 ± 3.6 (Celecoxib group). | | | | | |
|  | **Duration of CLBP, mean (range), y** | - 8.9 (8.8) (5 mg tanezumab group); - 7.7 (9.1) (10 mg tanezumab group). - 9.1 (10.5) (Celecoxib group). | | | | | |
|  | **Primary etiology, n (%)** | - Disc disease:32 (34.8) degenerative joint disease/OA: 13 (14.1) injury/muscular strain: 6 (6.5) other: 41 (44.6) (5 mg tanezumab group); - Disc disease:40 (43.0) degenerative joint disease/OA: 11 (11.8) injury/muscular strain: 0 other: 42 (45.2); - Disc disease:38 (41.3) degenerative joint disease/OA: 17 (18.5) injury/muscular strain: 1 (1.1) other: 36 (39.1) (Celecoxib group). | | | | | |
|  | **LBPI, mean ± SD** | - 6.74 ± 0.97 (5 mg tanezumab group); - 6.82 ± 1.09 (10 mg tanezumab group); - 6.72 ± 1.00 (Celecoxib group). | | | | | |
|  | **RMDQ, mean ± SD** | - 8.27 ± 5.02 (5 mg tanezumab group); - 8.12 ± 4.86 (10 mg tanezumab group); - 7.75 ± 4.95 (Celecoxib group). | | | | | |
| DATA for meta-analysis* (*: Only the arms that were included in meta-analyses are represented in this table.) | Continuous variables | Group | Sample size | Mean | Confidence Interval (Lower) | Confidence Interval (Upper) | Standard deviation |
|  | Δ LBPI at 2-week: 5 mg tanezumab versus NSAIDs | Tanezumab | 93 | -0.46 | -0.6 | -0.32 | 0.07 |
|  |  | NSAIDs | 92 | -0.45 | -0.59 | -0.31 | 0.07 |
|  | Δ LBPI at 4-week: 5 mg tanezumab versus NSAIDs | Tanezumab | 93 | -0.82 | -0.99 | -0.66 | 0.08 |
|  |  | NSAIDs | 92 | -0.55 | -0.73 | -0.38 | 0.09 |
|  | Δ LBPI at 8-week: 5 mg tanezumab versus NSAIDs | Tanezumab | 93 | -1.24 | -1.44 | -1.04 | 0.1 |
|  |  | NSAIDs | 92 | -1.07 | -1.28 | -0.86 | 0.11 |
|  | Δ LBPI at 12-week: 5 mg tanezumab versus NSAIDs | Tanezumab | 93 | -2.2 | -2.43 | -1.96 | 0.12 |
|  |  | NSAIDs | 92 | -1.85 | -2.08 | -1.62 | 0.12 |
|  | Δ LBPI at 16-week: 5 mg tanezumab versus NSAIDs | Tanezumab | 93 | -2.51 | -2.74 | -2.28 | 0.12 |
|  |  | NSAIDs | 92 | -2.29 | -2.54 | -2.06 | 0.12 |
|  | Δ LBPI at 1-week: 10 mg tanezumab versus NSAIDs | Tanezumab | 93 | -0.31 | -0.41 | -0.21 | 0.05 |
|  |  | NSAIDs | 92 | -0.19 | -0.32 | -0.12 | 0.05 |
|  | Δ LBPI at 2-week: 10 mg tanezumab versus NSAIDs | Tanezumab | 93 | -0.68 | -0.82 | -0.54 | 0.07 |
|  |  | NSAIDs | 92 | -0.45 | -0.59 | -0.31 | 0.07 |
|  | Δ LBPI at 4-week: 10 mg tanezumab versus NSAIDs | Tanezumab | 93 | -1.05 | -1.22 | -0.088 | 0.09 |
|  |  | NSAIDs | 92 | -0.55 | -0.71 | -0.35 | 0.09 |
|  | Δ LBPI at 8-week: 10 mg tanezumab versus NSAIDs | Tanezumab | 93 | -1.64 | -1.83 | -1.41 | 0.11 |
|  |  | NSAIDs | 92 | -1.07 | -1.24 | -0.79 | 0.11 |
|  | Δ LBPI at 12-week: 10 mg tanezumab versus NSAIDs | Tanezumab | 93 | -2.47 | -2.68 | -2.24 | 0.11 |
|  |  | NSAIDs | 92 | -1.85 | -2.08 | -1.6 | 0.12 |
|  | Δ LBPI at 16-week: 10 mg tanezumab versus NSAIDs | Tanezumab | 93 | -2.91 | -3.14 | -2.69 | 0.11 |
|  |  | NSAIDs | 92 | -2.29 | -2.4 | -1.92 | 0.12 |
|  | Δ RMQD at 2-week: 10 mg tanezumab versus NSAIDs | Tanezumab | 93 | -2.06 | -2.49 | -1.82 | 0.17 |
|  |  | NSAIDs | 92 | -1.2 | -1.53 | -0.87 | 0.17 |
|  | Δ RMQD at 4-week: 10 mg tanezumab versus NSAIDs | Tanezumab | 93 | -2.32 | -2.72 | -1.93 | 0.2 |
|  |  | NSAIDs | 92 | -1.87 | -2.26 | -1.48 | 0.2 |
|  | Δ RMQD at 8-week: 10 mg tanezumab versus NSAIDs | Tanezumab | 93 | -3.22 | -3.61 | -2.82 | 0.2 |
|  |  | NSAIDs | 92 | -2.64 | -3.06 | -2.28 | 0.2 |
|  | Δ RMQD at 16-week: 10 mg tanezumab versus NSAIDs | Tanezumab | 93 | -4.39 | -4.8 | -3.96 | 0.21 |
|  |  | NSAIDs | 92 | -1.07 | -1.25 | -0.81 | 0.11 |
|  | Dichotomous variables | Group | | Events | | Total | |
|  | Any AE: 5 mg tanezumab versus NSAIDs | Tanezumab | | 58 | | 92 | |
|  |  | NSAIDs | | 62 | | 92 | |
|  | Serious AE: 5 mg tanezumab versus NSAIDs | Tanezumab | | 4 | | 92 | |
|  |  | NSAIDs | | 2 | | 92 | |
|  | Treatment discontiuation: 5 mg tanezumab versus NSAIDs | Tanezumab | | 3 | | 92 | |
|  |  | NSAIDs | | 4 | | 92 | |
|  | Abonormal peripheral sensation: 5 mg tanezumab versus NSAIDs | Tanezumab | | 9 | | 92 | |
|  |  | NSAIDs | | 4 | | 92 | |
|  | Any AE: 10 mg tanezumab versus NSAIDs | Tanezumab | | 51 | | 93 | |
|  |  | NSAIDs | | 62 | | 92 | |
|  | Serious AE: 10 mg tanezumab versus NSAIDs | Tanezumab | | 9 | | 93 | |
|  |  | NSAIDs | | 2 | | 92 | |
|  | Treatment discontiuation: 10 mg tanezumab versus NSAIDs | Tanezumab | | 51 | | 93 | |
|  |  | NSAIDs | | 62 | | 92 | |
|  | Abonormal peripheral sensation: 10 mg tanezumab versus NSAIDs | Tanezumab | | 5 | | 93 | |
|  |  | NSAIDs | | 4 | | 92 | |
