# Supplementary material for: Comparison of tanezumab and non-steroidal anti-inflammatory drugs in efficacy and safety for chronic low back pain: a systematic review and meta-analysis of randomized controlled trials
Source: Front Neurol. 2025 Sep 1;16:1623280. doi: 10.3389/fneur.2025.1623280 (PMC12434963; doi:10.3389/fneur.2025.1623280)
Supplement: Supplementary file 3 [file Table_3.DOCX]

**Supplementary Table 3:** Risk of bias assessment.

1. Efficacy and safety of tanezumab in the treatment of chronic low back pain (Katz et al., 2011)

| RoB2 | Summary of bias considerations | Assessor (D.Y.) | Assessor (P.L.) | Date |
| --- | --- | --- | --- | --- |
| Randomization process | The study employed a robust randomization process, ensuring that participants were allocated to treatment groups (Tanezumab, Naproxen, or Placebo) in a 2:2:1 ratio. This methodological rigor helps to mitigate selection bias and provides a solid foundation for the study's internal validity. | Low risk | Low risk | 2025.3.17 |
| Deviations from the intended interventions | The study adhered strictly to the intended interventions, with participants receiving either a single intravenous infusion of Tanezumab or placebo, along with oral Naproxen or placebo. The protocol's adherence indicates a low risk of performance bias, suggesting that the interventions were administered as intended without significant deviations. | Low risk | Low risk | 2025.3.17 |
| Missing outcome data | The study included an intention-to-treat analysis, which incorporated all randomized participants, including those who did not complete the study. This comprehensive approach to data handling minimizes attrition bias and strengthens the study's ability to draw reliable conclusions from the data collected. | Low risk | Low risk | 2025.3.17 |
| Measurement of the outcome | Outcomes were measured using standardized and validated instruments. The use of these established tools ensures the accuracy and reliability of the outcome measurements, reducing measurement bias. | Low risk | Low risk | 2025.3.17 |
| Selection of the reported result | The study reported both primary and secondary outcome measures, demonstrating a commitment to transparency and completeness in the reporting of results. This practice minimizes reporting bias by ensuring that all relevant outcomes are presented, regardless of the direction or significance of the findings. | Low risk | Low risk | 2025.3.17 |

2. Efficacy and safety of tanezumab versus naproxen in the treatment of chronic low back pain (Kivitz et al., 2013)

| RoB2 | Summary of bias considerations | Assessor (D.Y.) | Assessor (P.L.) | Date |
| --- | --- | --- | --- | --- |
| Randomization process | The study was randomized, with patients allocated to treatment groups in a 2:2:3:3:3 ratio for placebo, tanezumab 5 mg, tanezumab 10 mg, tanezumab 20 mg, or naproxen. This method reduces selection bias and ensures a fair distribution of patients across groups. | Low risk | Low risk | 2025.3.17 |
| Deviations from the intended interventions | The study protocol was strictly followed, with all patients receiving their assigned treatments without significant deviations. This adherence to the intervention protocol minimizes performance bias. | Low risk | Low risk | 2025.3.17 |
| Missing outcome data | The study used an intent-to-treat analysis with baseline observation carried forward (BOCF) imputation for missing data, which helps to maintain the integrity of the data and reduces attrition bias. | Low risk | Low risk | 2025.3.17 |
| Measurement of the outcome | Outcomes were measured using standardized tools. These validated measures ensure accurate and consistent outcome assessment, reducing measurement bias. | Low risk | Low risk | 2025.3.17 |
| Selection of the reported result | The study reported both primary and secondary outcomes as pre-specified in the study protocol. There was no selective reporting of results, which maintains the integrity of the study findings and reduces reporting bias. | Low risk | Low risk | 2025.3.17 |

3. Tanezumab for chronic low back pain: a long-term, randomized, celecoxib controlled Japanese Phase III safety study (Konno et al., 2022)

| RoB2 | Summary of bias considerations | Assessor (D.Y.) | Assessor (P.L.) | Date |
| --- | --- | --- | --- | --- |
| Randomization process | The study employed a computer-generated blocked randomization scheme, assigning patients in a 1:1:1 ratio to treatment groups. This method ensures that each participant has an equal chance of receiving any of the study treatments, reducing selection bias. | Low risk | Low risk | 2025.3.17 |
| Deviations from the intended interventions | The study protocol included strict criteria for patient continuation past week 16, requiring a minimum reduction in LBPI score. This protocol adherence ensures that patients receiving continued treatment are those experiencing a therapeutic benefit, which could introduce a selection bias. However, this was a pre-specified criterion and thus is considered in the study design. | Low risk | Low risk | 2025.3.17 |
| Missing outcome data | The study used multiple imputation approaches to handle missing data, which is a robust method for addressing missing data and reducing potential attrition bias. | Low risk | Low risk | 2025.3.17 |
| Measurement of the outcome | Outcomes were measured using standardized tools. These validated measures ensure consistent and reliable outcome assessment across all treatment groups. | Low risk | Low risk | 2025.3.17 |
| Selection of the reported result | The study reported both primary safety outcomes and secondary efficacy outcomes as pre-specified in the study protocol. There was no indication of selective reporting, which maintains the integrity of the study findings. | Low risk | Low risk | 2025.3.17 |
